# Tertiary Lymphoid Structures Are Associated with Progression-Free Survival of Peripheral Neuroblastic Tumor Patients

**DOI:** 10.3390/cancers17081303

**Published:** 2025-04-12

**Authors:** Rebecca Rothe, Therés Golle, Basma Hachkar, Tina Hörz, Jessica Pablik, Luise Rupp, Ina Dietsche, Christian Kruppa, Guido Fitze, Marc Schmitz, Michael Haase, Rebekka Wehner

**Affiliations:** 1 Institute of Immunology, Faculty of Medicine Carl Gustav Carus, TUD Dresden University of Technology, 01307 Dresden, Germany; rebecca.rothe@nct-dresden.de (R.R.); theres.golle@tu-dresden.de (T.G.); basma.hachkar@uk-koeln.de (B.H.); luise.rupp@tu-dresden.de (L.R.); ina.dietsche@mailbox.tu-dresden.de (I.D.); marc.schmitz@tu-dresden.de (M.S.); 2 National Center for Tumor Diseases (NCT), NCT/UCC Dresden, a Partnership Between DKFZ, Faculty of Medicine and University Hospital Carl Gustav Carus, TUD Dresden University of Technology, and Helmholtz-Zentrum Dresden-Rossendorf (HZDR), 01307 Dresden, Germany; 3 German Cancer Consortium (DKTK), 01307 Dresden, Germany; 4 Department of Pediatric Surgery, University Hospital Carl Gustav Carus, 01307 Dresden, Germany; tina.hoerz@ukdd.de (T.H.); christian.kruppa@ukdd.de (C.K.); guido.fitze@ukdd.de (G.F.); michael.haase@ukdd.de (M.H.); 5 Department of Pathology, University Hospital Carl Gustav Carus, 01307 Dresden, Germany; jessica.pablik@ukdd.de

**Keywords:** tertiary lymphoid structures, T cells, macrophages, peripheral neuroblastic tumors, tumor immune microenvironment, multiplex immunohistochemistry

## Abstract

**Background/Objectives**: Peripheral neuroblastic tumors (pNT) are a biologically heterogeneous group of embryonal tumors that derive from the neural crest and affect the sympathetic nervous system. So far, little is known about the complex immune landscape in these rare childhood cancers. **Methods**: We focused on the immune cell infiltrate of treatment-naïve pNT from 24 patients, including high-risk neuroblastoma (HR-NBL), non-high-risk neuroblastoma (NHR-NBL), ganglioneuroblastoma (GNBL), and rare ganglioneuroma (GN). To gain novel insights into the immune architecture of these pNT subtypes, we used multiplex immunohistochemistry, multispectral imaging, and algorithm-based data evaluation to detect and characterize T cells, B cells, neutrophils, macrophages, and tertiary lymphoid structures (TLS). **Results**: The majority of the investigated tumor-infiltrating immune cells were macrophages and T cells. Their detailed phenotypic characterization revealed high proportions of M2-like macrophages as well as activated GrzB^+^ CD8^+^ and PD-1^+^ T lymphocytes. Proportions of these T cell phenotypes were significantly increased in GN compared to HR-NBL, NHR-NBL, or GNBL. In addition, TLS occurred in 11 of 24 patients, independent of immune cell frequencies in the whole tissues. Interestingly, all GN, most GNBL, but only a few NBL contained TLS. We distinguished between three TLS maturation stages that were present irrespective of the pNT subtype. The majority belonged to mature TLS of the primary follicle state. Mature LAMP3^+^ dendritic cells were also found, predominantly in T cell zones of TLS. Furthermore, TLS presence identified pNT patients with significantly prolonged progression-free survival in contrast to all other analyzed immunological features. **Conclusions**: We propose TLS to be a potential prognostic marker for pNT to predict patient outcomes.

## Simple Summary

Peripheral neuroblastic tumors (pNT) are a heterogeneous group of embryonal tumors. So far, little is known about the complex immune landscape in rare childhood cancers. We characterized T cells, B cells, macrophages, and tertiary lymphoid structures (TLS) in 24 treatment-naïve pNT patients, including neuroblastoma (NBL), ganglioneuroblastoma (GNBL), and rare ganglioneuroma (GN), using multiplex immunohistochemistry and algorithm-based data evaluation. The majority of tumor-infiltrating immune cells were macrophages and T cells. We found high proportions of M2-like macrophages and activated T lymphocytes, highlighting an increased immunological activity, especially in GN. TLS occurred in 11 of 24 patients, whereby all GN, most GNBL, but only a few NBL contained TLS. Further, we found three TLS maturation stages that were present in all pNT subtypes. We uncovered that TLS presence is associated with prolonged progression-free survival of pNT patients. Therefore, we propose that TLS are a potential prognostic marker for pNT to predict patient outcomes.

## 1. Introduction

Peripheral neuroblastic tumors (pNT) are a biologically heterogeneous group comprising a broad spectrum of clinical courses ranging from spontaneous regression without therapeutic interventions to tumors requiring intense therapy with sometimes fatal outcomes [1,2]. These pediatric tumors are neural crest-derived and affect the sympathetic nervous system [1,3,4,5]. They can be classified according to the International Neuroblastoma Pathology Classification (INPC) system as neuroblastoma (NBL), nodular or intermixed ganglioneuroblastoma (GNBL), as well as ganglioneuroma (GN) [6]. Histopathologically, NBL is characterized by undifferentiated or poorly differentiated neuroblasts and a minor amount of Schwannian stroma, whereas GN displays maturing or mature ganglion cells and is dominated by Schwannian stroma. GNBL shows mixed features of both NBL and GN subtypes [1,7]. Incidences of the rare pNT are given as about 10, <5, and 1 case(s) per million children for NBL, GNBL, and GN, respectively [8,9,10]. In particular, NBL is the most lethal solid extracranial pediatric malignancy, accounting for approximately 12–15% of cancer-related deaths in children [4]. On the contrary, GN is known to be benign and is more commonly diagnosed in older children (≥5 years of age) [1,11]. In many patients, pNT oncogenesis is driven by the amplification of protooncogene MYCN, which promotes karyorrhexis, as ranked by the mitosis–karyorrhexis index (MKI), as well as by segmental chromosome aberration [1,4]. These patient characteristics and tumor attributes are combined in the International Neuroblastoma Risk Group Staging System (INRGSS) [12], as well as prognostic grouping defining favorable histology and unfavorable histology [13].

Research deciphering the tumor immune microenvironment (TIME) has greatly improved the understanding of tumor establishment, progression, and its effect on therapies [14,15,16]. The interplay of the different immune cell subsets can support or inhibit cancer progression, whereby their phenotypic orientation has a crucial impact [16,17]. For instance, tumor-infiltrating macrophages can be divided into two main categories with distinct functions. M1-like macrophages are associated with inflammation and potential anti-tumorigenic immune responses. In contrast, M2-like macrophages display anti-inflammatory effector functions that support cancer cell survival [18,19,20]. Based on single-cell RNA-sequencing data, a recent study showed a higher expression of the M2-like macrophage marker CD163 in NBL compared to GNBL and GN samples [21]. Nevertheless, studies identifying macrophages based on protein levels in pNT are still rare. Within the TIME, macrophages and dendritic cells (DC) modulate the activity and function of T cells by the presentation of tumor antigens as well as stimulatory or inhibitory molecules [19,22,23,24]. Cancer antigen-specific CD8^+^ cytotoxic T cells efficiently lyse tumor cells, for example, via the release of granzyme B (GrzB) [25]. Moreover, CD4^+^ T helper cells promote anti-tumor immune responses by stimulating macrophages and DC as well as supporting CD8^+^ T cell and B cell activation [26,27]. In contrast, regulatory T cells (Treg) can promote tumor cell growth and survival [28,29]. T cell activation and function are modulated by cancer cells via the expression of ligands for co-inhibitory receptors, like programmed death 1 (PD-1) or lymphocyte activation gene 3 (LAG-3) [30,31]. These molecules also represent potential therapeutic targets already used in the immunotherapy of adult cancers [32]. In pNT, the majority of publications focused on T cell infiltration in NBL using flow cytometry or classical immunohistochemistry (IHC), as summarized by Wienke et al., while detailed phenotypic characterization or studies on GNBL and GN are rare [33]. Besides diffuse immune cell infiltrates, organized lymph node-like aggregates of T and B cells, so-called tertiary lymphoid structures (TLS), play a central role in the induction and maintenance of adaptive anti-tumor immune responses [34,35]. They provide the micromilieu to activate tumor antigen-specific CD4^+^ or CD8^+^ T cells and to promote B cell differentiation into plasma cells secreting tumor-reactive antibodies. TLS have been reported in a multitude of adult tumors and predominantly correlate with a beneficial clinical outcome of cancer patients [15,36,37,38,39]. However, TLS have been rarely studied in pediatric tumors so far, and little is known about their distribution and maturation. In pNT, organized lymphoid infiltrates were described in association with the opsoclonus-myoclonus syndrome (OMS) [40,41]. Besides OMS, TLS have been incidentally observed in earlier publications but without detailed characterization [42,43]. Therefore, we studied the TIME of high-risk neuroblastoma (HR-NBL), non-high-risk neuroblastoma (NHR-NBL), GNBL, and GN comprehensively by multiplex fluorescence-based IHC.

## 2. Materials and Methods

### 2.1. Patient Samples

This retrospective study includes a cohort of 24 tissue samples from children diagnosed with pNT between 2004 and 2021 at the Clinic and Policlinic for Pediatric Surgery of the University Hospital Carl Gustav Carus Dresden. The investigations were approved by the ethics committee of the TUD Dresden University of Technology (EK 378092017), and the donor’s parents (all patients were younger than 18 years of age) gave their written informed consent. The tumor tissues used were provided by the tissue bank for tumor and normal tissues (BioBank Dresden) at the National Center for Tumor Diseases Dresden (NCT/UCC) and the Institute for Pathology of the University Hospital Carl Gustav Carus Dresden. The samples were obtained from treatment-naïve primary tumors originating in adrenal glands, retroperitoneum, paravertebral regions, mediastinum, or abdomen and provided as 3 µm thin formalin-fixed and paraffin-embedded tissue slices. The clinical characteristics of all patients are summarized in Table 1.

### 2.2. Multiplex Immunohistochemistry

The different immune cell phenotypes comprising the TIME of human pNT were explored by multiplex IHC staining with a tyramide signal amplification approach. Stainings were performed using a Ventana Discovery Ultra Instrument (Roche, Basel, Switzerland) together with Opal™ multiplex reagents (Akoya Biosciences, Marlborough, MA, USA) as described previously [23,44,45,46]. Briefly, tissue slices were prepared by deparaffinization and rehydration in Discovery EZ Prep Solution (Roche) as well as heat-mediated antigen retrieval in ULTRA Cell Conditioning Solution 1 (CC1, Roche). Subsequently, primary antibodies (Abs) were applied manually and incubated at individual dilutions and time periods, as listed in Table S1. That was followed by incubation with appropriate secondary Abs (DISCOVERY OmniMap anti-mouse horseradish peroxidase (HRP) or OmniMap anti-rabbit HRP, both from Roche). Next, the Opal™ reagent containing one of the fluorophores Opal™ 520, 540, 570, 620, 650, or 690 (all Akoya Biosciences) was added manually. Prior to the subsequent staining cycle, all Abs were removed by heat-mediated Ab stripping in ULTRA Cell Conditioning Solution 2 (CC2, Roche). Staining sequences were repeated up to six times. In the final step, nuclei were counterstained with 4′,6-diamidino-2-phenylindole (DAPI; Sigma-Aldrich, St. Gallen, Switzerland). Then, tissue slices were coverslipped using Flouromount-G^®^ medium (SouthernBiotech, Birmingham, AL, USA) and stored at 4 °C protected from light until imaging. In total, stainings with four different protocols were implemented for visualization of the TIME composition. All utilized 19 primary Abs according to the different staining protocols, specific incubation temperatures, and times are listed in Table S1.

### 2.3. Image Acquisition and Analysis

Multispectral images of stained tissues were acquired using a Vectra^®^ 3.0 automated quantitative pathology imaging system (Akoya Biosciences). Overview scans of the whole tissues were taken with a 100× magnification, followed by the acquisition of regions of interest (ROIs) with a 200× magnification. To detect the immune cell infiltration or deciphering T cell and macrophage populations, ROIs were evenly distributed, covering 50–100% of the total tissue using Phenochart™ software (version 1.1; Akoya Biosciences). In order to analyze TLS, ROIs were set on cell aggregates based on DAPI signal density as well as T and B cell infiltration visible in the overview scans. After ROI acquisition, the single dyes of the multiplex images were separated by spectral unmixing with inForm^®^ software (versions 2.5 and 2.6; Akoya Biosciences).

Cells were quantified using trainable algorithms by employing inForm^®^ software. The algorithms were trained to discriminate between tissue and non-tissue regions, to separate single cells based on the DAPI signal, and to detect cell phenotypes by the characteristic staining pattern as well as the signal intensity of the respective marker molecules. Gained algorithms were validated in a separate set of training images and finally applied to all images. Data clearance was carried out to improve data quality. In a few cases, staining artifacts and insufficient signal intensities resulted in failed quality control requiring data exclusion that led to varying patient numbers in further data analysis. Obtained data were pre-processed using the R software (version 4.1.3; [47]) with phenoptr (version 0.3.2; [48]), phenoptrReports (version 0.3.2; [49]), and tidyverse (version 2.0.0, [50]) packages.

TLS maturation was evaluated as described in previous publications [45,51,52,53]. TLS regions were drawn manually, and their areas, as well as whole tissue areas, were calculated using inForm^®^ and QuPath software (version 0.4; [54]). In addition, LAMP3^+^ DC were counted manually and their cell densities within the single TLS areas were calculated. Representative images were prepared using Fiji software (version 2.9; [55]).

### 2.4. Gene Set Variation Analysis

From GEO database (https://www.ncbi.nlm.nih.gov/geo/, accessed on 23 February 2024) and ArrayExpress Archive (http://www.ebi.ac.uk/microarray-as/ae/, accessed on 23 February 2024), gene expression datasets of NBL, GNBL, and GN samples were obtained (GSE7529, GSE12460, GSE16476, GSE18271, E-MEXP-83). Of note, limited GNBL and GN patient numbers and information about pNT differentiation according to INRGSS, MYCN status, or neoadjuvant treatments were not available in every dataset. In order to analyze the enrichment of TLS-associated gene signatures in these datasets, we conducted a gene set variation analysis using the GSVA package (version 1.50.5; [56]) in R (version 4.3.0). We selected 83 TLS-related genes (Table S2) according to recent publications [57,58], and the median of these genes was referred to as the TLS enrichment score for each sample.

### 2.5. Statistical Analysis

Data was statistically evaluated using GraphPad Prism (version 9.5; GraphPad Software, Boston, MA, USA). Prior to further analysis, the data were tested for normal distribution. Mann–Whitney test and Kruskal–Wallis test, followed by Dunn’s multiple comparisons test as post hoc analysis, were implemented to compare two groups or perform multiple group comparisons. All *p*-values ≤ 0.05 were considered statistically significant. Individual dot plot graphs depict median values with a 95% confidence interval (CI). Bar plots were created using R software (version 4.1.3; [47]) by employing the plotly package (version 4.10.2; [59]). Cluster analysis was implemented using R software with the ComplexHeatmap package (version 2.15.4; [60]). For this purpose, cell frequencies were utilized, and data were normalized by empirical percentile transformation. The Ward method was applied for hierarchical clustering. In terms of visualization of publically available datasets, expression data of the selected 83 TLS-related genes were clustered using the pheatmap package (version 1.0.12; [61]) with Euclidean distance for clustering columns and rows. For correlation analysis, frequencies of different immune cell phenotypes (B cells, T cells, and macrophages), normalized TLS area, and amount of Schwannian stroma were used, and nonparametric Spearman r as well as two-tailed *p*-values with 95% CI were calculated by GraphPad Prism.

## 3. Results

### 3.1. pNT Subtypes Differ in Their T Cell but Not in Their Macrophage Compartment

**Figure 1:**
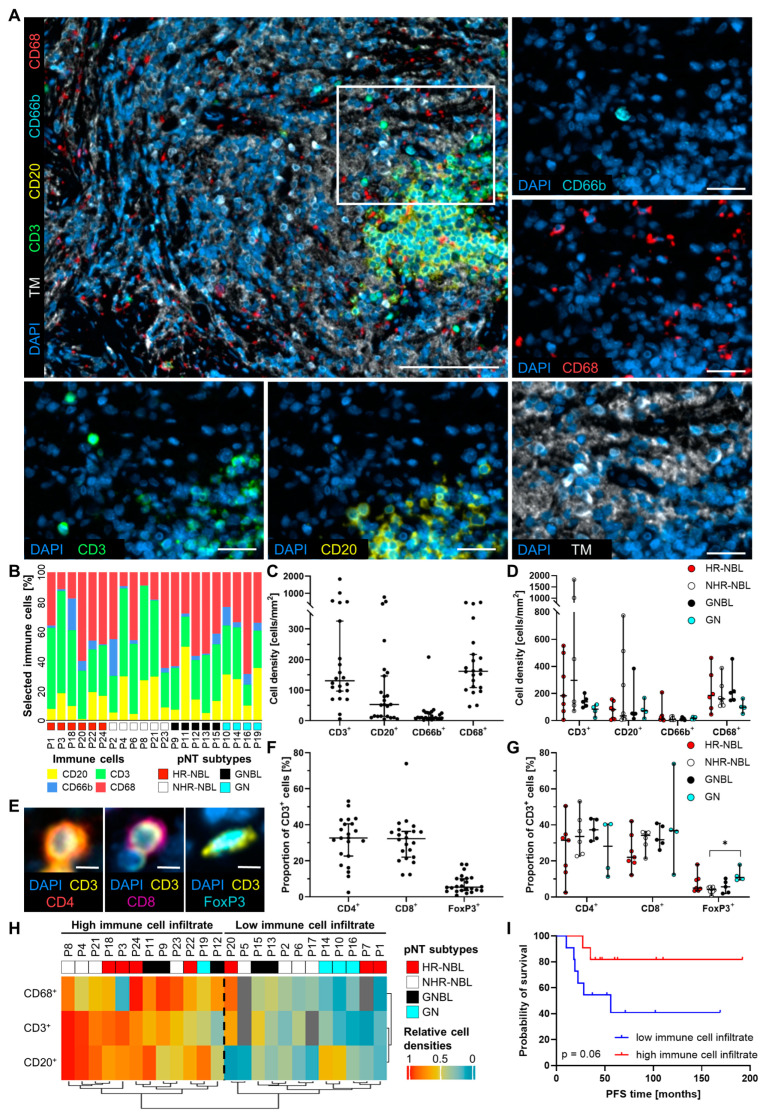
Overview of the general immune cell infiltrates in human pNT. (**A**) Representative multiplex IHC images visualize tumor cells (tumor marker [TM]: synaptophysin/CD56, white), T cells (CD3, green), B cells (CD20, yellow), neutrophils (CD66b, cyan), and macrophages (CD68, red). Nuclei were stained with DAPI (blue). Scale bars indicate 100 µm (merge image) and 25 µm (magnified image). (**B**) Bar plot shows the proportion of CD3^+^, CD20^+^, CD66b^+^, and CD68^+^ immune cells for the individual HR-NBL, NHR-NBL, GNBL, and GN patients. (**C**) Quantified densities of CD3^+^, CD20^+^, CD66b^+^, and CD68^+^ cells are depicted for all 24 pNT tissues investigated and (**D**) pNT subtypes HR-NBL, NHR-NBL, GNBL, and GN. (**E**) Representative multiplex IHC images illustrate CD4^+^ CD3^+^ (T helper cells, red/yellow), CD8^+^ CD3^+^ (cytotoxic T cells, magenta/yellow), and FoxP3^+^ CD3^+^ (Treg, cyan/yellow). Scale bars indicate 5 µm. (**F**) Calculated proportions of T cells in all pNT tissues and (**G**) classified in pNT subtypes are shown in dot plots. Median with 95% confidence interval (CI); Mann–Whitney test; * *p* ≤ 0.05. (**H**) The heatmap created by unsupervised clustering presents the stratification of HR-NBL, NHR-NBL, GNBL, and GN patients in a low and a high immune cell infiltrate cluster. (**I**) Kaplan–Meier curves show progression-free survival (PFS) for pNT patients stratified based on their high/low immune cell infiltrate. *p*-values calculated by Log-rank test.

To gain novel insights into the composition of the pNT microenvironment, the immune cell infiltrate was explored in 24 tumor samples consisting of seven HR-NBL, eight NHR-NBL, five GNBL, and four rare GN (Table 1). We analyzed treatment-naïve tumors to exclude therapy-related changes in the TIME. CD3^+^ T cells, CD20^+^ B lymphocytes, CD68^+^ macrophages, CD66b^+^ neutrophils, the nucleus-defining dye DAPI, and tumor cells expressing CD56 and synaptophysin (tumor markers: TM) were stained by multi-parametric fluorescence IHC together in one tissue section (Figure 1A). Macrophages and T cells infiltrated the Schwannian stroma and areas of neuroblasts with varying degrees of differentiation. In addition, lymphocytes formed aggregates of varying sizes and composition. B lymphocytes were found in aggregates, usually in conjunction with T cells. In contrast, neutrophils were mainly localized in vessels. Figure 1B shows the heterogeneous distribution of the immune cell populations across individual pNT patients, as summarized in Figure 1C. Macrophages (161.6 cells/mm^2^, 95% CI 138.7 to 244.6 cells/mm^2^) represent the majority of immune cells, followed by T cells (130.5 cells/mm^2^, 95% CI 107.0 to 474.7 cells/mm^2^). We detected fewer B cells (52.5 cells/mm^2^, 95% CI 46.8 to 205.2 cells/mm^2^) and some neutrophils (9.9 cells/mm^2^, 95% CI 5.1 to 39.6 cells/mm^2^). We found no significant differences in the frequency of tumor-infiltrating immune cells among the four investigated groups. However, a trend indicated lower macrophage and T cell frequencies in GN compared to HR-NBL, NHR-HBL, or GNBL (Figure 1D). Further, CD3^+^ T lymphocytes were distinguished into CD4^+^ T helper cells, CD8^+^ T cells, and FoxP3^+^ CD3^+^ T cells (Figure 1E). Calculated proportions of these T cell populations demonstrated an almost equal infiltration of CD4^+^ (32.6%, 95% CI 25.5 to 37.1%) and CD8^+^ (32.3%, 95% CI 25.8 to 37.2%) T cells and a markedly lower proportion of FoxP3^+^ CD3^+^ T cells (5.4%, 95% CI 5.0 to 9.1%) (Figure 1F). However, T cell subtypes did not show strong variations between HR-NBL, NHR-NBL, and GNBL. Only FoxP3^+^ CD3^+^ T cell proportions were significantly higher in GN compared to the NHR-NBL group (Figure 1G). Moreover, we clustered the 24 pNT patients based on the overall infiltration of CD3^+^ T cells, CD20^+^ B lymphocytes, and CD68^+^ macrophages (Figure 1H), resulting in two groups (low and high immune cell infiltrate) significantly differing in the densities of T and B lymphocytes as well as macrophages (Figure S1A). However, there were hardly any differences with regard to the composition of the subpopulations and phenotypes of the individual immune cells (Figure S1B–F). Concerning pNT subtypes, the clustering approach did not show a clear association of the immune cell infiltrate with HR-NBL, NHR-NBL, GNBL, or GN, as each of the four pNT subtypes was represented in both clusters (Figure 1H). In order to further analyze the prognostic relevance of immune cell infiltration, the overall survival and progression-free survival (PFS) of pNT patients in our cohort were calculated (Table 1). In general, 91% of the patients survived, with a follow-up ranging from 35 to 208 months. However, 36.4% of our pNT patients showed progression within 10 to 56 months. In particular, HR-NBL and nodular GNBL patients demonstrated unfavorable PFS compared to NHR-NBL and intermixed GNBL, respectively (Figure S2A). Separating pNT patients according to the high and low immune cell cluster, a prolonged PFS for highly infiltrated pNT patients was demonstrated compared to the “low immune cell infiltrate” group (*p* = 0.06, Figure 1I). In particular, all intermixed GNBL samples were highly infiltrated by immune cells, while nodular GNBL clustered in the “low immune cell infiltrate” group. Furthermore, the two HR-NBL and NHR-NBL patients of our pNT cohort, who died 45 or 50 months after diagnosis (Table 1), belonged to the “low immune cell infiltrate” group, underlining an association of immune cell infiltration with survival.

**Figure 2:**
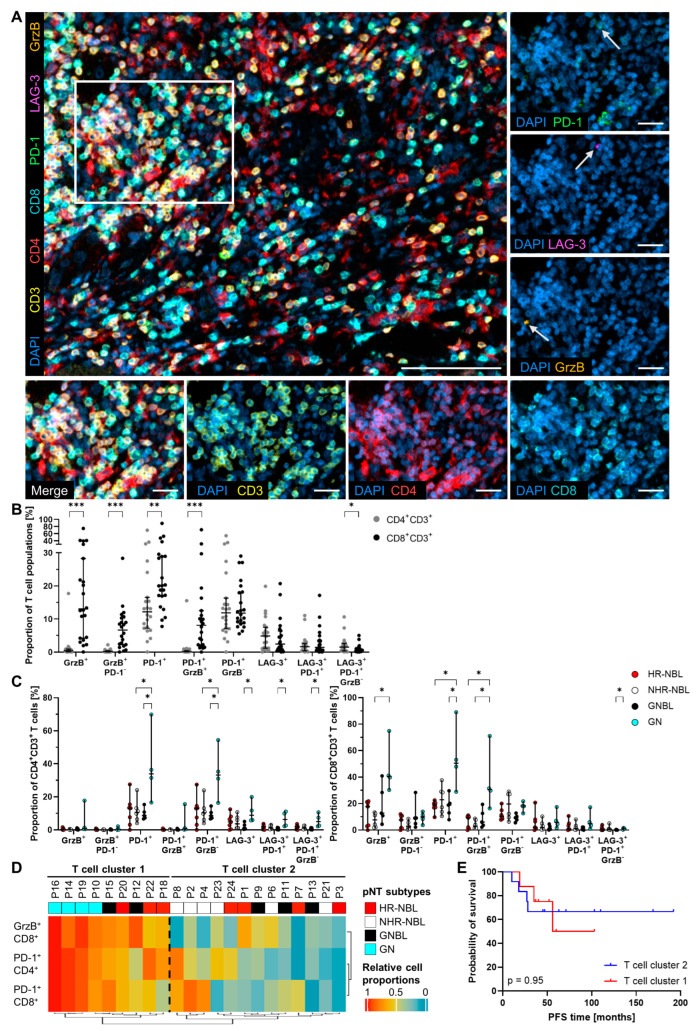
Detailed phenotypic characterization of T cell populations infiltrating pNT tissue samples. (**A**) Representative image of a pNT section demonstrates tissue infiltration by CD3^+^ (yellow), CD4^+^ (red), or CD8^+^ (cyan) T cells expressing inhibitory receptors PD-1 (green), LAG-3 (magenta), and/or cytotoxic marker GrzB (orange). Nuclei were stained with DAPI (blue). PD-1^+^, LAG-3^+^, and GrzB^+^ cells are highlighted by white arrows. Scale bars indicate 100 µm (merge image) and 25 µm (magnified area). (**B**) Calculated proportions of T helper (CD4^+^ CD3^+^) and cytotoxic T cell (CD8^+^ CD3^+^) populations defined by the (co-)expression (+) or absence (−) of investigated markers as presented in the graph. (**C**) CD4^+^ CD3^+^ (left) and CD8^+^ CD3^+^ T cell proportions (right) distributed to HR-NBL, NHR-NBL, GNBL, and GN are shown in dot plots. Median with 95% confidence interval (CI); Mann–Whitney test (**B**) and Kruskal–Wallis test with Dunn‘s multiple comparisons post hoc test (**C**); * *p* ≤ 0.05, ** *p* ≤ 0.01, *** *p* ≤ 0.001. (**D**) The heatmap created by unsupervised clustering presents the clustering of patients with high and low active T cell phenotype infiltration. (**E**) Kaplan–Meier curves demonstrate PFS for pNT patients stratified according to their high/low infiltration of active T cell phenotypes. *p*-values calculated by Log-rank test.

To determine whether certain phenotypes of macrophages or T cells have an impact on patient outcome, we used multi-parametric IHC stainings to detect the immune checkpoint molecules PD-1 and LAG-3 as well as the cytotoxic molecule GrzB on CD4^+^ or CD8^+^ T cells (Figure 2A). Among the analyzed marker molecules, PD-1 was most abundantly expressed by both T cell populations (PD-1^+^ CD4^+^/PD-1^+^ CD8^+^, Figure 2B). A significantly higher proportion of CD8^+^ T cells expressed GrzB compared to CD4^+^ T lymphocytes. In particular, more than half of the GrzB^+^ CD8^+^ T cells co-expressed PD-1. In contrast, the proportions of GrzB^−^ PD-1^+^ cells among CD4^+^ and CD8^+^ lymphocytes were almost equal. Furthermore, we found a low but increased percentage of inhibitory receptor LAG-3^+^ T cells within the CD4^+^ (4.9%, 95% CI 3.1 to 7.5%) compared to the CD8^+^ (2.4%, 95% CI 2.1 to 7.0%) T cell subset. A closer look at these LAG-3^+^ T cells revealed that just a minor fraction co-expressed PD-1. The percentage of the LAG-3^+^ PD-1^+^ GrzB^−^ population, defining exhausted lymphocytes, was low but significantly enhanced among CD4^+^ T cells. In addition, T cells infiltrating HR-NBL, NHR-NBL, and GNBL showed a similar phenotype (Figure 2C). In GN, the percentages of PD-1^+^ T lymphocytes, GrzB^+^ CD8^+^ T cells, and LAG-3^+^ CD4^+^ T cells were significantly enhanced, indicating a more active T cell compartment in GN compared to the other three pNT subtypes. This is in line with a clustering approach based on relevant T cell phenotypes (Figure 2D). Here, all GN patients are characterized by high proportions of GrzB^+^ CD8^+^, PD-1^+^ CD4^+^, or PD-1^+^ CD8^+^, defining T cell clusters 1 and 2. Nevertheless, T cell frequencies did not affect the PFS of pNT patients, as shown for these T cell clusters combining relevant T cell phenotypes and individual CD4^+^ and CD8^+^ T cell phenotypes (Figure 2E and Figure S2B).

**Figure 3:**
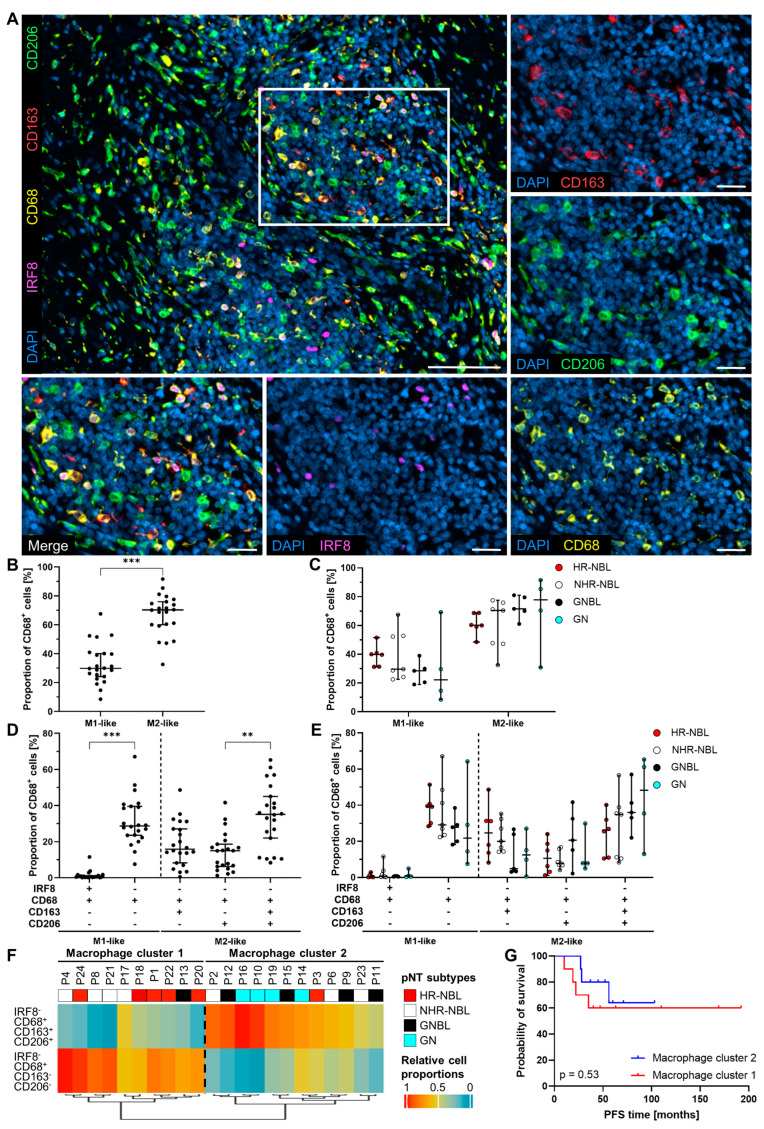
Investigation of pNT TIME regarding macrophage infiltration and polarization. (**A**) Representative images of a pNT section visualize stained marker molecules IRF8 (M1, magenta), CD68 (pan-macrophage marker, yellow), CD163 (M2, red), and CD206 (M2, green). Nuclei were stained with DAPI (blue). Scale bars indicate 100 µm (merge image) and 25 µm (magnified area). (**B**) Calculated M1- and M2-like macrophage proportions of all CD68^+^ cells are given as dot plots for all pNT samples and (**C**) HR-NBL, NHR-NBL, GNBL, and GN patients. (**D**) Calculated proportions of dissected M1-like (IRF8^−^ CD68^+^ CD163^−^ CD206^−^ and IRF8^+^ CD68^+^ CD163^−^ CD206^−^) and M2-like (IRF8^−^ CD68^+^ CD163^+^ CD206^−^, IRF8^−^ CD68^+^ CD206^+^ CD163^−^, and IRF8^−^ CD68^+^ CD163^+^ CD206^+^) macrophage subpopulations are displayed for all pNT patients and (**E**) pNT subtypes. Positive marker expression (+) or absent marker expression (−) was defined for each investigated marker as displayed below the axes. Median with 95% confidence interval (CI); Mann–Whitney test (**B**) and Kruskal–Wallis test with Dunn‘s multiple comparisons post hoc test (**D**); ** *p* ≤ 0.01, *** *p* ≤ 0.001. (**F**) The heatmap created by unsupervised clustering shows the stratification of patients based on single CD68^+^ (M1-like) and triple CD68^+^CD163^+^CD206^+^ (M2-like) macrophages. (**G**) Kaplan–Meier curves demonstrate PFS for pNT patients stratified based on the unsupervised clustering of the two above-mentioned macrophage populations. *p*-values calculated by Log-rank test.

Besides T cells, macrophages frequently infiltrated pNT. Therefore, we performed additional multiplex IHC stainings to discriminate macrophage subpopulations with regard to their polarization. We supplemented the general macrophage marker CD68 with nuclear transcription factor interferon regulatory factor (IRF) 8, scavenger receptor CD163, and mannose receptor CD206 in order to achieve a more precise detection of M1-like and M2-like macrophage subtypes [62,63,64,65] (Figure 3A). We calculated the proportions of M1-like (single CD68^+^ or double IRF8^+^ CD68^+^, with both M1-like subtypes being defined negative for CD163 and CD206) and M2-like (double CD68^+^ CD163^+^, double CD68^+^ CD206^+^ or triple CD68^+^ CD163^+^ CD206^+^, with all three M2-like subtypes being defined negative for IRF8) macrophages (Figure 3B). The data showed higher percentages of M2-like (70.2%, 95% CI 60.0 to 75.9%) than M1-like (29.8%, 95% CI 24.2 to 40.0%) macrophages in the whole cohort as well as in HR-NBL, NHR-NBL, GNBL, and GN (Figure 3B,C). A more detailed dissection of macrophage populations demonstrated nuclear IRF8 expression by a small fraction of M1-like macrophages (Figure 3D). Among M2-like macrophages, most of the CD68^+^ cells co-expressed both CD163 and CD206. The distribution of the macrophage subpopulations among HR-NBL, NHR-NBL, GNBL, and GN patients did not reveal significant differences (Figure 3E). Clustering pNT patients based on the dominant macrophage subpopulations resulted in two clusters with either high single CD68^+^ (cluster 1) or high triple CD68^+^ CD163^+^ CD206^+^ cell frequencies (cluster 2, Figure 3F). Of note, the four GN were highly infiltrated by triple CD68^+^ CD163^+^ CD206^+^ macrophages and were all found in cluster 2. However, the PFS of pNT patients grouped according to single CD68^+^ and triple CD68^+^ CD163^+^ CD206^+^ macrophage clusters or abundancies of individual macrophage subpopulations did not differ significantly (Figure 3G and Figure S2C).

### 3.2. TLS Are a Common Component of pNT with Variable Presence in HR-NBL, NHR-NBL, GNBL, and GN Independent of Their Maturation

**Figure 4:**
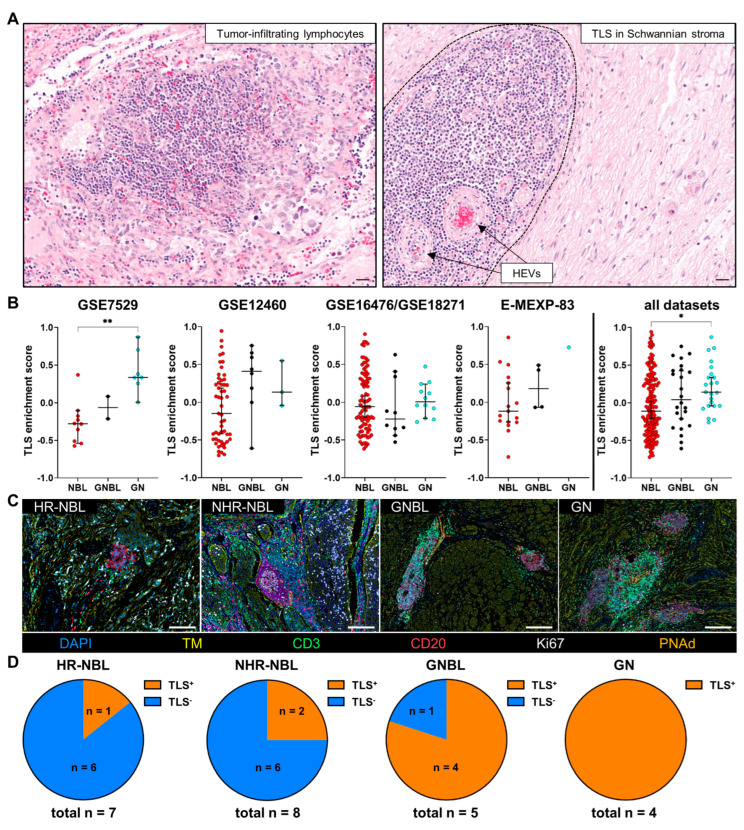
Frequency of TLS in NBL, GNBL, and GN samples. (**A**) Representative H&E images of pNT tissues show a lymphocyte aggregates in the tumor area (left) and a clearly defined, mature TLS (framed by a black dashed line) in the Schwannian stroma containing two HEVs marked by arrows (right). Scale bars indicate 50 µm. (**B**) Dot plots demonstrate the median enrichment scores of 83 TLS-related gene signatures calculated by gene set variation analysis (GSVA) using publically available gene expression datasets. Median with 95% confidence interval (CI); Kruskal–Wallis test with Dunn‘s multiple comparisons post hoc test; * *p* ≤ 0.05, ** *p* ≤ 0.01. (**C**) Representative multiplex IHC images of TLS in tissue sections of patients diagnosed with HR-NBL, NHR-NBL, GNBL, and GN. Scale bars indicate 200 µm. (**D**) Pie charts visualize the distribution of TLS-containing tissues among pNT subtypes.

As mentioned before, we found T and B cells aggregated in dense cell clusters as a first hint of the TLS presence. H&E stainings (Figure 4A) confirmed the localization of loose lymphocyte aggregates within the tumor cell areas as well as in the Schwannian stroma, whereas structured TLS were predominantly embedded in the Schwannian stroma compartment. In order to get first insights into TLS presence within pNT samples, we analyzed gene expression data obtained by microarray experiments from publically available databases regarding the occurrence of TLS-related gene signatures in NBL, GNBL, and GN tumor samples (Figure 4B). In all gene datasets, the amount of pNT subtypes classified according to INPC reflected the higher frequency of NBL (168 samples) and the rarity of GNBL (24 samples) and GN (23 samples) similar to our cohort. By GSVA, the calculated TLS enrichment score represented the median expression of 83 TLS-related genes [57,58] in each sample and each dataset (Figure 4B and Figure S3). Although two datasets (GSE7529, E-MEXP-83) demonstrated a clear enrichment of TLS-related gene signatures in GN compared to the other pNT subtypes, no significantly different TLS enrichment scores were calculated for the other datasets (GSE12460, GSE16476/GSE18271). However, looking at the combined TLS enrichment scores of all evaluated GEO datasets demonstrated a significant association between TLS-related gene expression and histologically defined INPC classes. To verify the trend of enriched TLS-related gene signatures in our pNT cohort, multiplex IHC Abs against CD3, CD20, Ki67, peripheral node addressin (PNAd), and a combination of synaptophysin, CD56, and chromogranin A as TM, were used to define TLS and their maturation stages in the tissue sections (Figure 4C). We detected TLS in 11 patients out of the 24 analyzed pNT tissues. An assignment according to the pNT subtypes demonstrated that all GN, four out of five GNBL, two out of eight NHR-NBL, and only one out of seven HR-NBL contained TLS (Figure 4D). This indicates that TLS presence might be reduced with the degree of malignancy.

**Figure 5:**
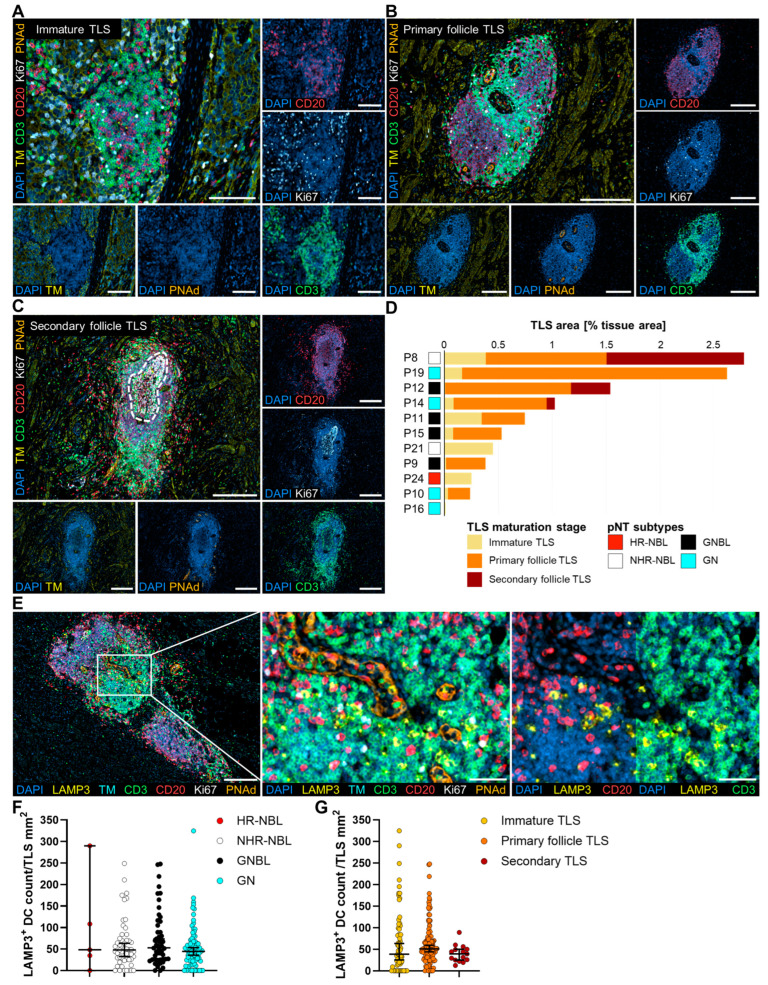
Multiplex immunohistochemical characterization of TLS maturation and LAMP3^+^ DC infiltration. Representative images of an immature TLS (**A**), a primary follicle TLS (**B**), and a secondary follicle TLS (**C**) stained for tumor marker (TM: synaptophysin/chromogranin A/CD56; yellow), CD3 (T cells, green), CD20 (B cells, red), Ki67 (proliferating cells, white), and PNAd (high endothelial venules (HEVs), orange). Nuclei were detected with DAPI (blue). The germinal center (GC) of secondary follicle TLS is highlighted by a white dashed line. Scale bars indicate 100 µm (**A**), 200 µm (**B**), and 300 µm (**C**). (**D**) Bar plot shows areas of immature TLS, primary follicle TLS, or secondary follicle TLS normalized to the whole tissue areas of the corresponding individual pNT patients. (**E**) Representative images highlight LAMP3^+^ cells (mature DC, yellow) infiltration in a primary follicle TLS (TM: synaptophysin/chromogranin A/CD56; cyan), CD3 (T cells, green), CD20 (B cells, red), Ki67 (proliferating cells, white), and PNAd (high endothelial venules (HEVs), orange). Scale bars indicate 200 µm in the overview images and 50 µm in the magnified areas. Densities of LAMP3^+^ DC in individual TLS are shown for (**F**) HR-NBL, NHR-NBL, GNBL, GN, and (**G**) TLS maturation stages. Median with 95% confidence interval (CI).

Moreover, we dissected the maturation stages of TLS in pNT (Figure 5A–D). TLS were manually evaluated according to a classification scheme adopted from previous descriptions [45,51,52,53,66]. Dense aggregates consisting of T and B cells with limited spatial organization were considered immature TLS (Figure 5A). Cell clusters with distinct T and B cell zones and/or high endothelial venules (HEVs) reflected mature TLS (Figure 5B,C). Furthermore, mature TLS stages were separated into primary or secondary follicle TLS based on the absence or presence of Ki67^+^ CD20^+^ B cells defining the germinal center (GC, Figure 5B,C). The majority of TLS were assigned as primary follicles and were found in 8 of the 11 TLS^+^ pNT with varying counts up to 43 primary follicle TLS per tissue (Figure S4A). Secondary follicle TLS was detected in three patients who also exhibited primary follicle and immature TLS, whereby one patient exhibited 12 TLS at each maturation stage. Concerning the TLS area, the highest TLS proportions of total tissue area were found in pNT patients with secondary follicle TLS and many primary follicle TLS (>10 primary follicle TLSs per tissue, Figure 5D). Three patients contained only immature TLS. In general, the different TLS maturity stages did not correlate with pNT subtypes, as the three tissues with mature, secondary follicle TLS belonged to GN, GNBL, and NHR-NBL. In addition, we stained and counted mature LAMP3^+^ DC in a single TLS (Figure 5E). Of note, LAMP3^+^ DC infiltrated TLS via HEVs and was found mainly in the T cell zones of TLS, interacting with plenty T cells and a few B cells. However, the density of LAMP3^+^ DC did not differ significantly among the four pNT subtypes or three TLS maturation stages (Figure 5F,G), as LAMP3^+^ DC count correlated significantly with the TLS area (Figure S4B). For instance, immature TLS having the smallest TLS areas contained the least LAMP3^+^ DC (Figure S4C,D).

### 3.3. Presence of TLS Is Linked to Prolonged Progression-Free Survival of pNT Patients

**Figure 6:**
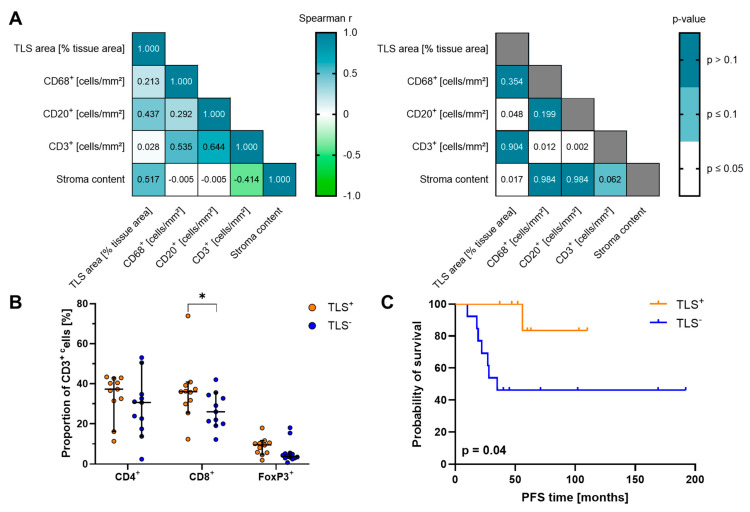
Association of relevant immune cell populations and TLS to patient characteristics and tumor attributes. (**A**) Correlation matrix comprising TLS area, densities of tumor-infiltrated CD3^+^, CD20^+^, and CD68^+^ cells, as well as proportions of FoxP3^+^ CD3^+^ T cells and cytotoxic CD8^+^ T cells in conjunction with Schwannian stroma content and patient age at diagnosis was build showing Spearman r correlation coefficient (left) and corresponding *p*-values (right). (**B**) Calculated proportions of CD4^+^, CD8^+^ as well as FoxP3^+^ CD3^+^ T cells are demonstrated for TLS^+^ and TLS^−^ groups. Median with 95% confidence interval (CI); * *p* ≤ 0.05 (**C**) Kaplan–Meier curves show PFS for pNT patients stratified based on the presence/absence of TLS. *p*-values calculated by Log-rank test.

Furthermore, we performed correlation approaches combining our relevant immune cell data with important histopathological tumor attributes, such as Schwannian stroma content of pNT patients (Figure 6A). Looking at the results with calculated nonparametric Spearman r and *p*-values, significant positive correlations between CD20^+^ B cells and CD3^+^ T cell densities or TLS area were obvious, underlining the organization of TLS in distinct B and T cell zones. T cell density was also positively associated with CD68^+^ macrophage infiltration. Moreover, Schwannian stroma content correlated significantly with the TLS area, strengthening the above-mentioned observations in terms of TLS localization. Based on TLS presence in the tumor tissues, we separated our pNT cohort into a TLS^+^ and a TLS^−^ subgroup and re-evaluated immune cell infiltration. The densities of CD3^+^ T cells, CD66b^+^ neutrophils, and CD68^+^ macrophages, proportions of T cell phenotypes co-expressing PD-1, LAG-3, and/or GrzB as well as M1- and M2-like macrophages were almost equal in both TLS groups (Figure 6A and Figure S5). As expected from the correlation analysis, a significantly higher number of CD20^+^ B cells was observed in the TLS^+^ group (TLS^+^: 78.89 cells/mm^2^, 95% CI 14.52 to 774.09 cells/mm^2^; TLS^−^: 13.92 cells/mm^2^, 95% CI 4.68 to 512.97 cells/mm^2^) most likely caused by the B cell zones of TLS. Among T lymphocytes, only proportions of CD8^+^ T cells were significantly enhanced in TLS^+^ patients (Figure 6B). As frequencies of CD20^+^ B and CD8^+^ T cells were significantly increased in TLS^+^ pNT tissues, both immune cell populations were tested with respect to their impact on patients’ survival (Figure S5F,G). However, the individual immune cell populations did not affect PFS significantly. Interestingly, TLS^+^ pNT patients showed a significantly improved PFS in comparison to TLS^−^ patients (*p* = 0.04, Figure 6C). As all GN and intermixed GNBL contained TLS and both pNT subtypes are considered more benign tumors, we looked at NBL patients only and found that the presence of TLS was also linked to favorable PFS in HR-NBL and NHR-NBL patients compared to their respective counterparts (Figure S5H). Therefore, the presence of TLS, as an important immunological feature, has a particular impact on tumor progression of pNT patients.

## 4. Discussion

Over the last decades, numerous studies extensively characterized the composition and spatial organization of the TIME in adult cancers highlighting the importance of the immune cell infiltrate for tumor development, progression, clinical outcome, and therapeutic response [16,25]. Less well understood is the immune architecture of pediatric tumors that harbor fundamental differences, e.g. a lower mutational burden or a generally reduced immune cell infiltration [6,67,68]. To fill this gap, we analyzed the TIME of the different pNT subtypes HR-NBL, NHR-NBL, GNBL, and GN. In addition, conflicting data exist concerning TIME characterization or T cell infiltration in pNT and the clinical outcomes of patients [69,70,71]. These divergent results, mainly raised by transcriptomic data evaluation, might be caused by varying patient cohort compositions, including results from therapy-naïve and differently pre-treated samples [43,72,73,74]. Therefore, we selected a well-defined patient cohort with treatment-naïve primary tumor tissues to reduce confounders based on tumor localization and treatment strategies.

The immune cell proportions of the pNT patients in our cohort mirror the heterogeneity within and among the pNT subtypes published earlier [21,43,75]. In addition, our findings confirmed single-cell RNA-sequencing data defining macrophages and T cells as the most frequent immune cell types [21,43]. The enhanced frequency of M2-like macrophages was also reported by previous investigations using either transcriptome or IHC data focusing on NBL patients [43,72,74,76,77]. Yuan et al. compared subtypes of pNT based on single-cell RNA-sequencing data and demonstrated that NBL samples showed a higher expression of the M2-like macrophage marker CD163 compared to GNBL and GN samples [21]. The authors suggested a potential pro-tumorigenic microenvironment in NBL as opposed to non-NBL. Our data confirmed the dominance of M2-like macrophages but revealed no significant differences in the distribution of macrophage subpopulations separated by pNT subsets, TLS occurrence, or overall immune cell infiltrate. Additional analysis demonstrated that CD68^+^ macrophage infiltration correlated with the frequency of CD3^+^ T cells, indicating a potential cross-talk between these two immune cell populations. We also detected CD4^+^, CD8^+^ T cells, and Treg in the tissues of our pNT cohort, as other studies focusing on NBL [43,75]. Although, we found increased proportions of CD8^+^ T cells in tissues of pNT patients containing TLS an association of CD8^+^ T cells with PFS of pNT patients was not detected. This is in contrast to publications demonstrating a positive impact of CD3^+^, CD4^+^, or CD8^+^ T cells on an improved survival and prognosis of NBL patients [75,78]. In our cohort, Schwannian stroma-rich GN tumors displayed the largest median FoxP3^+^ CD3^+^ T cell proportions. Mina et al. also found an association of high FoxP3 expression in fibrovascular septa regions surrounding tumor nests in NBL samples [75], partially supporting our results. Furthermore, to evaluate the phenotype and cytotoxic capacity of T cells within pNT in more detail, we analyzed the expression of PD-1, LAG-3, and GrzB. Proportions of GrzB^+^ and/or PD-1^+^ CD8^+^ T cells were significantly enhanced in GN compared to HR-NBL, NHR-NBL, or GNBL. This highlighted the activated state of CD8^+^ T cells infiltrating benign GN. However, these T cell phenotypes were not associated with prolonged PFS. In addition, we demonstrated that a very small fraction of PD-1^+^ T cells also presented LAG-3 as a sign of exhaustion, whereby PD-1^+^ LAG-3^+^ CD4^+^ T cells were mainly found in GN as well. However, the extent of potentially exhausted T cells is different to our own data in adult head and neck squamous cell carcinoma. Here, we used a similar staining protocol and found high proportions of exhausted PD-1^+^ and/or LAG-3^+^ CD8^+^ T cells compared to single PD-1^+^ once [79]. In contrast to our findings, PD-1 expression on NBL-infiltrating lymphocytes was associated with a good disease outcome [80,81].

In our cohort, the frequency of CD3^+^ T lymphocytes correlated significantly positive with CD20^+^ B cells. B cells were significantly more abundant in TLS-containing tissues, but CD20^+^ density did not serve as a prognostic marker for PFS in our pNT patients. Nevertheless, we detected TLS in 11 of 24 analyzed pNT tissues, confirming data for OMS NBL patients [82]. In our cohort, TLS were found in all GN, most GNBL, two NHR-NBL, and one HR-NBL. Thus, TLS occurrence might be reduced with increasing malignancy. Until now, TLS in pNT were distinguished from loose lymphocyte aggregates using H&E staining or fluorescence-based IHC of CD3, CD20, and Ki67 [40,41,82]. In this context, Chen et al. observed TLS in alveolar (*n* = 10 out of 22) and embryonal (*n* = 9 out of 25) rhabdomyosarcomas using IHC stainings of CD3 and CD20 to define TLS [83]. To the best of our knowledge, we classified TLS maturation stages for the first time in pNT. We distinguished between immature TLS and mature, primary, or secondary follicle TLS based on the occurrence of HEVs, GC, as well as organized B and T cell zones. Independent of the respective pNT subtypes, we identified all TLS maturation stages in our cohort, whereby primary follicle TLS were the most frequent ones. In addition, LAMP3^+^ DC counts in the single TLS correlated with the corresponding TLS areas, whereby the smallest immature TLS were the least frequently infiltrated ones. In the literature, infiltration of LAMP3^+^ DC, representing a mature, antigen-presenting DC population, is linked to TLS maturation stages, as early TLS had a significantly lower abundance of LAMP3^+^ DC than primary or secondary follicle TLS in clear cell renal cell carcinoma [84]. In addition, the presence of TLS was not only associated with significantly increased proportions of CD8^+^ T cells and CD20^+^ B lymphocytes but also correlated with Schwannian stroma. According to the literature, the content of Schwannian stroma is discussed as a potentially prognostic marker and is thought to be linked to tumor cell differentiation [85,86]. In line with our study, Kasikova et al. reported significantly higher CD8^+^ T cell densities in TLS^+^ high-grade serous ovarian cancer tissues compared to TLS^−^ ones [87]. Moreover, analyzing TLS with multiplex IHC in human papilloma virus-negative head and neck squamous cell carcinoma, Li et al. found significantly higher frequencies of CD20^+^ B cells, CD8^+^ T cells, and LAMP3^+^ DC in TLS^+^ tissues than in TLS^−^ tumor samples [88], consistently supporting our findings. Although there was no distinct correlation of TLS^+^ tissues with the phenotypically characterized T cell and macrophage subpopulations, we clearly demonstrated that the presence of TLS was significantly associated with prolonged PFS, confirming published data for adult tumors, like breast cancer, non-small cell lung cancer, colorectal cancer or melanoma [14,28,89,90,91]. Furthermore, Morcrette et al. hypothesized that chemotherapy-induced TLS formation resulted in a good prognosis of adenomatous polyposis coli (APC) germline-mutated hepatoblastoma children [66]. Despite the limited sample size of rare pNT tissues, our study paves the way for additional functional studies on TLS or investigations explaining their formation in pediatric tumors. These results might support the design of novel TLS-addressing therapies for pNT patients.

## 5. Conclusions

In contrast to adult tumors, the general immune cell infiltrate did not significantly influence the classification and PFS of pNT patients. However, we found enhanced proportions of activated T cells associated with benign GN. We identified TLS in all maturation stages as a common component of pNT, with TLS presence being linked to reduced tumor malignancy and prolonged PFS of pNT patients. Therefore, we propose investigating TLS presence in order to improve the prediction of a patient’s prognosis.

## Figures and Tables

**Table 1:** Clinical characteristics of pNT patients.

PatientID	Gender	Age at Diagnosis (Months)	INPC	Differentiation Stage	Stroma (%)	INRGSS Group	MYCN	MKI	Histology	Survival	OS Time (Months)	Progress	PFS Time (Months)	TLS Count
P1	f	174	NBL	poorly differentiated	5	high	-	low	UH	alive	169	-	169	0
P2	m	33	NBL	differentiating	30	intermediate	-	low	FH	dead	50	+	28	0
P3	m	48	NBL	poorly differentiated	5	high	-	low	UH	alive	208	+	27	0
P4	m	20	NBL	poorly differentiated	30	low	-	intermediate	UH	alive	192	-	192	0
P5	m	2	NBL	poorly differentiated	10	intermediate	-	low	FH	alive	102	-	102	0
P6	m	1	NBL	poorly differentiated	3	low	-	low	FH	alive	71	-	71	0
P7	m	41	NBL	poorly differentiated	5	high	-	low	UH	dead	45	+	18	0
P8	m	12	NBL	poorly differentiated	3	intermediate	-	low	FH	alive	110	-	110	36
P9	f	28	GNBL	intermixed	80	low	-	n.a.	FH	n.a.	n.a.	n.a.	n.a.	11
P10	m	149	GN	mature	99	low	-	n.a.	FH	alive	56	+	56	4
P11	f	33	GNBL	intermixed	40	low	-	low	FH	alive	103	-	103	7
P12	m	65	GNBL	intermixed	99	low	-	low	FH	alive	103	-	103	18
P13	f	128	GNBL	nodular	7	intermediate	-	low	UH	alive	93	+	10	0
P14	m	85	GN	maturing	95	low	-	n.a.	FH	alive	52	-	52	20
P15	f	83	GNBL	nodular	45	intermediate	-	low	UH	alive	37	-	37	14
P16	m	127	GN	maturing	99	low	n.a.	n.a.	FH	n.a.	n.a.	n.a.	n.a.	2
P17	m	12	NBL	differentiating	10	intermediate	-	low	FH	alive	63	+	22	0
P18	f	23	NBL	poorly differentiated	7	high	-	low	UH	alive	40	-	40	0
P19	m	74	GN	maturing	99	low	n.a.	n.a.	FH	alive	60	-	60	52
P20	m	63	NBL	poorly differentiated	7	high	-	high	UH	alive	35	+	19	0
P21	f	5	NBL	poorly differentiated	7	low	-	intermediate	FH	alive	63	-	63	17
P22	m	39	NBL	undifferentiated	0	high	-	n.a.	UH	alive	45	+	35	0
P23	m	7	NBL	poorly differentiated	40	low	-	low	FH	alive	45	-	45	0
P24	f	47	NBL	poorly differentiated	40	high	-	low	UH	alive	47	-	47	5

Female (f); male (m); not available (n.a.); negative (-); positive (+); favorable histology (FH); unfavorable histology (UH); overall survival (OS: time to death or last follow up); progression-free survival (PFS: time to relapse/progress or last follow up).

## Data Availability

The original contributions presented in the study are included in the article/Supplementary Material. Further inquiries can be directed to the corresponding author.

## Supplementary Materials

The following supporting information can be downloaded at https://www.mdpi.com/article/10.3390/cancers17081303/s1, Figure S1. Immune cell frequencies in pNT patients clustered according to low and high immune cell infiltration. Figure S2. Survival analysis of individual T cell phenotypes and macrophage subpopulations. Figure S3. Heatmaps depict gene expression data of 83 TLS-related genes for four publically available microarray datasets using Euclidean distance for clustering columns and rows. Figure S4. Analysis of TLS count per tissue and LAMP3^+^ DC in single TLS. Figure S5. Immune cell frequencies based on TLS presence/absence in pNT tissue samples. Table S1. List of 19 antibodies and reagents used for multiplex IHC. Table S2. List of TLS-related genes.

## References

[1] Choi J.H., Ro J.Y. (2022). Mediastinal Neuroblastoma, Ganglioneuroblastoma, and Ganglioneuroma: Pathology Review and Diagnostic Approach. Semin. Diagn. Pathol..

[2] Shimada H., Sano H., Hazard F.K. (2020). Pathology of Peripheral Neuroblastic Tumors. Clin. Pediatr. Hematol. Oncol..

[3] Brodeur G.M. (2003). Neuroblastoma: Biological Insights into a Clinical Enigma. Nat. Rev. Cancer.

[4] Johnsen J.I., Dyberg C., Wickström M. (2019). Neuroblastoma—A Neural Crest Derived Embryonal Malignancy. Front. Mol. Neurosci..

[5] Körber V., Stainczyk S.A., Kurilov R., Henrich K.-O., Hero B., Brors B., Westermann F., Höfer T. (2023). Neuroblastoma Arises in Early Fetal Development and Its Evolutionary Duration Predicts Outcome. Nat. Genet..

[6] Pfister S.M., Reyes-Múgica M., Chan J.K.C., Hasle H., Lazar A.J., Rossi S., Ferrari A., Jarzembowski J.A., Pritchard-Jones K., Hill D.A. (2022). A Summary of the Inaugural WHO Classification of Pediatric Tumors: Transitioning from the Optical into the Molecular Era. Cancer Discov..

[7] Park J.R., Eggert A., Caron H. (2010). Neuroblastoma: Biology, Prognosis, and Treatment. Hematol./Oncol. Clin. N. Am..

[8] Colon N.C., Chung D.H. (2011). Neuroblastoma. Adv. Pediatr..

[9] Radhakrishnan S., Rao R., Lashkari H.P., Kini H., Kini J.R., Kudurugundi V.B., Ashok V., Gowthuvalli C.V. (2023). A Needle in the Haystack: An Unusual Case Presentation of Ganglioneuroblastoma at a Tertiary Care Center in Coastal Karnataka. Clin. Case Rep..

[10] Shah D., Chaudhary S.R., Khan S., Mallik S. (2023). Overreliance on Radiological Findings Leading to Misdiagnosed Giant Retroperitoneal Ganglioneuroma: A Case Report and Literature Review. Cureus.

[11] Conces M.R. (2020). Peripheral Neuroblastic Tumors of the Adrenal Gland: Clinicopathologic Features and Important Molecular Alterations. Diagn. Histopathol..

[12] Irwin M.S., Naranjo A., Zhang F.F., Cohn S.L., London W.B., Gastier-Foster J.M., Ramirez N.C., Pfau R., Reshmi S., Wagner E. (2021). Revised Neuroblastoma Risk Classification System: A Report From the Children’s Oncology Group. J. Clin. Oncol..

[13] Shimada H., Ambros I.M., Dehner L.P., Hata J., Joshi V.V., Roald B., Stram D.O., Gerbing R.B., Lukens J.N., Matthay K.K. (1999). The International Neuroblastoma Pathology Classification (the Shimada System). Cancer.

[14] Binnewies M., Roberts E.W., Kersten K., Chan V., Fearon D.F., Merad M., Coussens L.M., Gabrilovich D.I., Ostrand-Rosenberg S., Hedrick C.C. (2018). Understanding the Tumor Immune Microenvironment (TIME) for Effective Therapy. Nat. Med..

[15] Fridman W.H., Zitvogel L., Sautès–Fridman C., Kroemer G. (2017). The Immune Contexture in Cancer Prognosis and Treatment. Nat. Rev. Clin. Oncol..

[16] Galon J., Bruni D. (2020). Tumor Immunology and Tumor Evolution: Intertwined Histories. Immunity.

[17] Mackenzie N.J., Nicholls C., Templeton A.R., Perera M.P., Jeffery P.L., Zimmermann K., Kulasinghe A., Kenna T.J., Vela I., Williams E.D. (2022). Modelling the Tumor Immune Microenvironment for Precision Immunotherapy. Clin. Transl. Immunol..

[18] Cendrowicz E., Sas Z., Bremer E., Rygiel T.P. (2021). The Role of Macrophages in Cancer Development and Therapy. Cancers.

[19] Italiani P., Boraschi D. (2014). From Monocytes to M1/M2 Macrophages: Phenotypical vs. Functional Differentiation. Front. Immunol..

[20] Liu J., Geng X., Hou J., Wu G. (2021). New Insights into M1/M2 Macrophages: Key Modulators in Cancer Progression. Cancer Cell Int..

[21] Yuan X., Seneviratne J.A., Du S., Xu Y., Chen Y., Jin Q., Jin X., Balachandran A., Huang S., Xu Y. (2022). Single-Cell Profiling of Peripheral Neuroblastic Tumors Identifies an Aggressive Transitional State That Bridges an Adrenergic-Mesenchymal Trajectory. Cell Rep..

[22] Duan Z., Luo Y. (2021). Targeting Macrophages in Cancer Immunotherapy. Signal Transduct. Target. Ther..

[23] Plesca I., Benešová I., Beer C., Sommer U., Müller L., Wehner R., Heiduk M., Aust D., Baretton G., Bachmann M.P. (2022). Clinical Significance of Tumor-Infiltrating Conventional and Plasmacytoid Dendritic Cells in Pancreatic Ductal Adenocarcinoma. Cancers.

[24] Truxova I., Kasikova L., Hensler M., Skapa P., Laco J., Pecen L., Belicova L., Praznovec I., Halaska M.J., Brtnicky T. (2018). Mature Dendritic Cells Correlate with Favorable Immune Infiltrate and Improved Prognosis in Ovarian Carcinoma Patients. J. ImmunoTherapy Cancer.

[25] Bruni D., Angell H.K., Galon J. (2020). The Immune Contexture and Immunoscore in Cancer Prognosis and Therapeutic Efficacy. Nat. Rev. Cancer.

[26] Pereira M.V.A., Galvani R.G., Gonçalves-Silva T., De Vasconcelo Z.F.M., Bonomo A. (2024). Tissue Adaptation of CD4 T Lymphocytes in Homeostasis and Cancer. Front. Immunol..

[27] Speiser D.E., Chijioke O., Schaeuble K., Münz C. (2023). CD4+ T Cells in Cancer. Nat. Cancer.

[28] Kang W., Feng Z., Luo J., He Z., Liu J., Wu J., Rong P. (2021). Tertiary Lymphoid Structures in Cancer: The Double-Edged Sword Role in Antitumor Immunity and Potential Therapeutic Induction Strategies. Front. Immunol..

[29] Kumagai S., Itahashi K., Nishikawa H. (2024). Regulatory T Cell-Mediated Immunosuppression Orchestrated by Cancer: Towards an Immuno-Genomic Paradigm for Precision Medicine. Nat. Rev. Clin. Oncol..

[30] Andrews L.P., Marciscano A.E., Drake C.G., Vignali D.A.A. (2017). LAG 3 (CD 223) as a Cancer Immunotherapy Target. Immunol. Rev..

[31] Patsoukis N., Wang Q., Strauss L., Boussiotis V.A. (2020). Revisiting the PD-1 Pathway. Sci. Adv..

[32] Roy D., Gilmour C., Patnaik S., Wang L.L. (2023). Combinatorial Blockade for Cancer Immunotherapy: Targeting Emerging Immune Checkpoint Receptors. Front. Immunol..

[33] Wienke J., Dierselhuis M.P., Tytgat G.A.M., Künkele A., Nierkens S., Molenaar J.J. (2021). The Immune Landscape of Neuroblastoma: Challenges and Opportunities for Novel Therapeutic Strategies in Pediatric Oncology. Eur. J. Cancer.

[34] Chen Y., Wu Y., Yan G., Zhang G. (2024). Tertiary Lymphoid Structures in Cancer: Maturation and Induction. Front. Immunol..

[35] Teillaud J.-L., Houel A., Panouillot M., Riffard C., Dieu-Nosjean M.-C. (2024). Tertiary Lymphoid Structures in Anticancer Immunity. Nat. Rev. Cancer.

[36] Fridman W.H., Meylan M., Pupier G., Calvez A., Hernandez I., Sautès-Fridman C. (2023). Tertiary Lymphoid Structures and B Cells: An Intratumoral Immunity Cycle. Immunity.

[37] Jacquelot N., Tellier J., Nutt S., Belz B. (2021). Tertiary Lymphoid Structures and B Lymphocytes in Cancer Prognosis and Response to Immunotherapies. OncoImmunology.

[38] Sautès-Fridman C., Petitprez F., Calderaro J., Fridman W.H. (2019). Tertiary Lymphoid Structures in the Era of Cancer Immunotherapy. Nat. Rev. Cancer.

[39] Schumacher T.N., Thommen D.S. (2022). Tertiary Lymphoid Structures in Cancer. Science.

[40] Cooper R., Khakoo Y., Matthay K.K., Lukens J.N., Seeger R.C., Stram D.O., Gerbing R.B., Nakagawa A., Shimada H. (2001). Opsoclonus-Myoclonus-Ataxia Syndrome in Neuroblastoma: Histopathologic Features-A Report from the Children’s Cancer Group. Med. Pediatr. Oncol..

[41] Gambini C., Conte M., Bernini G., Angelini P., Pession A., Paolucci P., Donfrancesco A., Veneselli E., Mazzocco K., Tonini G.P. (2003). Neuroblastic Tumors Associated with Opsoclonus-Myoclonus Syndrome: Histological, Immunohistochemical and Molecular Features of 15 Italian Cases. Virchows Arch..

[42] Melaiu O., Chierici M., Lucarini V., Jurman G., Conti L.A., De Vito R., Boldrini R., Cifaldi L., Castellano A., Furlanello C. (2020). Cellular and Gene Signatures of Tumor-Infiltrating Dendritic Cells and Natural-Killer Cells Predict Prognosis of Neuroblastoma. Nat. Commun..

[43] Verhoeven B.M., Mei S., Olsen T.K., Gustafsson K., Valind A., Lindström A., Gisselsson D., Fard S.S., Hagerling C., Kharchenko P.V. (2022). The Immune Cell Atlas of Human Neuroblastoma. Cell Rep. Med..

[44] Kießler M., Plesca I., Sommer U., Wehner R., Wilczkowski F., Müller L., Tunger A., Lai X., Rentsch A., Peuker K. (2021). Tumor-Infiltrating Plasmacytoid Dendritic Cells Are Associated with Survival in Human Colon Cancer. J. Immunother. Cancer.

[45] Rupp L., Dietsche I., Kießler M., Sommer U., Muckenhuber A., Steiger K., Van Eijck C.W.F., Richter L., Istvanffy R., Jäger C. (2024). Neoadjuvant Chemotherapy Is Associated with Suppression of the B Cell-Centered Immune Landscape in Pancreatic Ductal Adenocarcinoma. Front. Immunol..

[46] Rupp L., Resag A., Potkrajcic V., Warm V., Wehner R., Jöhrens K., Bösmüller H., Eckert F., Schmitz M. (2023). Prognostic Impact of the Post-Treatment T Cell Composition and Spatial Organization in Soft Tissue Sarcoma Patients Treated with Neoadjuvant Hyperthermic Radio(Chemo)Therapy. Front. Immunol..

[47] R Core Team (2023). R: A Language and Environment for Statistical Computing.

[48] Johnson K.S. (2022). Phenoptr: InForm Helper Functions.

[49] Johnson K.S. (2022). Phenoptrreports: Create Reports Using Phenoptics Data 2022.

[50] Wickham H., Averick M., Bryan J., Chang W., McGowan L., François R., Grolemund G., Hayes A., Henry L., Hester J. (2019). Welcome to the Tidyverse. J. Open Source Softw..

[51] Fridman W.H., Meylan M., Petitprez F., Sun C.-M., Italiano A., Sautès-Fridman C. (2022). B Cells and Tertiary Lymphoid Structures as Determinants of Tumour Immune Contexture and Clinical Outcome. Nat. Rev. Clin. Oncol..

[52] Gunderson A.J., Rajamanickam V., Bui C., Bernard B., Pucilowska J., Ballesteros-Merino C., Schmidt M., McCarty K., Philips M., Piening B. (2021). Germinal Center Reactions in Tertiary Lymphoid Structures Associate with Neoantigen Burden, Humoral Immunity and Long-Term Survivorship in Pancreatic Cancer. OncoImmunology.

[53] Sun X., Liu W., Sun L., Mo H., Feng Y., Wu X., Li C., Chen C., Li J., Xin Y. (2022). Maturation and Abundance of Tertiary Lymphoid Structures Are Associated with the Efficacy of Neoadjuvant Chemoimmunotherapy in Resectable Non-Small Cell Lung Cancer. J. Immunother. Cancer.

[54] Bankhead P., Loughrey M.B., Fernández J.A., Dombrowski Y., McArt D.G., Dunne P.D., McQuaid S., Gray R.T., Murray L.J., Coleman H.G. (2017). QuPath: Open Source Software for Digital Pathology Image Analysis. Sci. Rep..

[55] Schindelin J., Arganda-Carreras I., Frise E., Kaynig V., Longair M., Pietzsch T., Preibisch S., Rueden C., Saalfeld S., Schmid B. (2012). Fiji: An Open-Source Platform for Biological-Image Analysis. Nat. Methods.

[56] Hänzelmann S., Castelo R., Guinney J. (2013). GSVA: Gene Set Variation Analysis for Microarray and RNA-Seq Data. BMC Bioinform..

[57] Du W., Huang X., Liu R., Ye F., Li X., Sun B., Li H. (2024). Transcriptome Analysis of Tertiary Lymphoid Structures (TLSs)-Related Genes Reveals Prognostic Value and Immunotherapeutic Potential in Cancer. Oncologie.

[58] Wu Z., Zhou J., Xiao Y., Ming J., Zhou J., Dong F., Zhou X., Xu Z., Zhao X., Lei P. (2022). CD20+CD22+ADAM28+ B Cells in Tertiary Lymphoid Structures Promote Immunotherapy Response. Front. Immunol..

[59] Sievert C. (2020). Interactive Web-Based Data Visualization with R, Plotly, and Shiny.

[60] Gu Z. (2022). Complex Heatmap Visualization. iMeta.

[61] Kolde R. (2024). Pheatmap: Pretty Heatmaps, version 1.0 12.

[62] Chistiakov D.A., Myasoedova V.A., Revin V.V., Orekhov A.N., Bobryshev Y.V. (2018). The Impact of Interferon-Regulatory Factors to Macrophage Differentiation and Polarization into M1 and M2. Immunobiology.

[63] Gao J., Liang Y., Wang L. (2022). Shaping Polarization Of Tumor-Associated Macrophages In Cancer Immunotherapy. Front. Immunol..

[64] Günthner R., Anders H.-J. (2013). Interferon-Regulatory Factors Determine Macrophage Phenotype Polarization. Mediat. Inflamm..

[65] Yao Y., Xu X.-H., Jin L. (2019). Macrophage Polarization in Physiological and Pathological Pregnancy. Front. Immunol..

[66] Morcrette G., Hirsch T.Z., Badour E., Pilet J., Caruso S., Calderaro J., Martin Y., Imbeaud S., Letouzé E., Rebouissou S. (2019). *APC* Germline Hepatoblastomas Demonstrate Cisplatin-Induced Intratumor Tertiary Lymphoid Structures. OncoImmunology.

[67] Holterhus M., Altvater B., Kailayangiri S., Rossig C. (2022). The Cellular Tumor Immune Microenvironment of Childhood Solid Cancers: Informing More Effective Immunotherapies. Cancers.

[68] Sherif S., Roelands J., Mifsud W., Ahmed E.I., Raynaud C.M., Rinchai D., Sathappan A., Maaz A., Saleh A., Ozer E. (2022). The Immune Landscape of Solid Pediatric Tumors. J. Exp. Clin. Cancer Res..

[69] Terry R.L., Meyran D., Ziegler D.S., Haber M., Ekert P.G., Trapani J.A., Neeson P.J. (2020). Immune Profiling of Pediatric Solid Tumors. J. Clin. Investig..

[70] Masih K.E., Wei J.S., Milewski D., Khan J. (2021). Exploring and Targeting the Tumor Immune Microenvironment of Neuroblastoma. J. Cell. Immunol..

[71] Anderson J., Majzner R.G., Sondel P.M. (2022). Immunotherapy of Neuroblastoma: Facts and Hopes. Clin. Cancer Res..

[72] Batchu S. (2021). Immunological Landscape of Neuroblastoma and Its Clinical Significance. Cancer Treat. Res. Commun..

[73] Feng C., Li T., Xiao J., Wang J., Meng X., Niu H., Jiang B., Huang L., Deng X., Yan X. (2022). Tumor Microenvironment Profiling Identifies Prognostic Signatures and Suggests Immunotherapeutic Benefits in Neuroblastoma. Front. Cell Dev. Biol..

[74] Wienke J., Visser L.L., Kholosy W.M., Keller K.M., Barisa M., Poon E., Munnings-Tomes S., Himsworth C., Calton E., Rodriguez A. (2024). Integrative Analysis of Neuroblastoma by Single-Cell RNA Sequencing Identifies the NECTIN2-TIGIT Axis as a Target for Immunotherapy. Cancer Cell.

[75] Mina M., Boldrini R., Citti A., Romania P., D’Alicandro V., De Ioris M., Castellano A., Furlanello C., Locatelli F., Fruci D. (2015). Tumor-Infiltrating T Lymphocytes Improve Clinical Outcome of Therapy-Resistant Neuroblastoma. OncoImmunology.

[76] Asgharzadeh S., Salo J.A., Ji L., Oberthuer A., Fischer M., Berthold F., Hadjidaniel M., Liu C.W.-Y., Metelitsa L.S., Pique-Regi R. (2012). Clinical Significance of Tumor-Associated Inflammatory Cells in Metastatic Neuroblastoma. J. Clin. Oncol..

[77] Larsson K., Kock A., Idborg H., Arsenian Henriksson M., Martinsson T., Johnsen J.I., Korotkova M., Kogner P., Jakobsson P.-J. (2015). COX/mPGES-1/PGE _2_ Pathway Depicts an Inflammatory-Dependent High-Risk Neuroblastoma Subset. Proc. Natl. Acad. Sci. USA.

[78] Schaafsma E., Jiang C., Cheng C. (2021). B Cell Infiltration Is Highly Associated with Prognosis and an Immune-Infiltrated Tumor Microenvironment in Neuroblastoma. J. Cancer Metastasis Treat..

[79] Kirchner J., Plesca I., Rothe R., Resag A., Löck S., Benešová I., Rupp L., Linge A., Wehner R., Krause M. (2024). Type I Conventional Dendritic Cells and CD8+ T Cells Predict Favorable Clinical Outcome of Head and Neck Squamous Cell Carcinoma Patients. Front. Immunol..

[80] Chowdhury F., Dunn S., Mitchell S., Mellows T., Ashton-Key M., Gray J.C. (2015). PD-L1 and CD8 ^+^ PD1 ^+^ Lymphocytes Exist as Targets in the Pediatric Tumor Microenvironment for Immunomodulatory Therapy. OncoImmunology.

[81] Melaiu O., Mina M., Chierici M., Boldrini R., Jurman G., Romania P., D’Alicandro V., Benedetti M.C., Castellano A., Liu T. (2017). PD-L1 Is a Therapeutic Target of the Bromodomain Inhibitor JQ1 and, Combined with HLA Class I, a Promising Prognostic Biomarker in Neuroblastoma. Clin. Cancer Res..

[82] Rosenberg M.I., Greenstein E., Buchkovich M., Peres A., Santoni-Rugiu E., Yang L., Mikl M., Vaksman Z., Gibbs D.L., Reshef D. (2023). Polyclonal Lymphoid Expansion Drives Paraneoplastic Autoimmunity in Neuroblastoma. Cell Rep..

[83] Chen L., Oke T., Siegel N., Cojocaru G., Tam A.J., Blosser R.L., Swailes J., Ligon J.A., Lebid A., Morris C. (2020). The Immunosuppressive Niche of Soft-Tissue Sarcomas Is Sustained by Tumor-Associated Macrophages and Characterized by Intratumoral Tertiary Lymphoid Structures. Clin. Cancer Res..

[84] Xu W., Lu J., Liu W.-R., Anwaier A., Wu Y., Tian X., Su J.-Q., Qu Y.-Y., Yang J., Zhang H. (2023). Heterogeneity in Tertiary Lymphoid Structures Predicts Distinct Prognosis and Immune Microenvironment Characterizations of Clear Cell Renal Cell Carcinoma. J. Immunother. Cancer.

[85] Weiss T., Taschner-Mandl S., Janker L., Bileck A., Rifatbegovic F., Kromp F., Sorger H., Kauer M.O., Frech C., Windhager R. (2021). Schwann Cell Plasticity Regulates Neuroblastic Tumor Cell Differentiation via Epidermal Growth Factor-like Protein 8. Nat. Commun..

[86] Gomez R.L., Ibragimova S., Ramachandran R., Philpott A., Ali F.R. (2022). Tumoral Heterogeneity in Neuroblastoma. Biochim. Biophys. Acta (BBA)—Rev. Cancer.

[87] Kasikova L., Rakova J., Hensler M., Lanickova T., Tomankova J., Pasulka J., Drozenova J., Mojzisova K., Fialova A., Vosahlikova S. (2024). Tertiary Lymphoid Structures and B Cells Determine Clinically Relevant T Cell Phenotypes in Ovarian Cancer. Nat. Commun..

[88] Li H., Zhu S.-W., Zhou J.-J., Chen D.-R., Liu J., Wu Z.-Z., Wang W.-Y., Zhang M.-J., Sun Z.-J. (2023). Tertiary Lymphoid Structure Raises Survival and Immunotherapy in HPV—HNSCC. J. Dent. Res..

[89] Di Caro G., Bergomas F., Grizzi F., Doni A., Bianchi P., Malesci A., Laghi L., Allavena P., Mantovani A., Marchesi F. (2014). Occurrence of Tertiary Lymphoid Tissue Is Associated with T-Cell Infiltration and Predicts Better Prognosis in Early-Stage Colorectal Cancers. Clin. Cancer Res..

[90] Cabrita R., Lauss M., Sanna A., Donia M., Skaarup Larsen M., Mitra S., Johansson I., Phung B., Harbst K., Vallon-Christersson J. (2020). Tertiary Lymphoid Structures Improve Immunotherapy and Survival in Melanoma. Nature.

[91] Dieu-Nosjean M.-C., Antoine M., Danel C., Heudes D., Wislez M., Poulot V., Rabbe N., Laurans L., Tartour E., De Chaisemartin L. (2008). Long-Term Survival for Patients With Non–Small-Cell Lung Cancer With Intratumoral Lymphoid Structures. J. Clin. Oncol..

