# Supplementary material for: Tertiary Lymphoid Structures Are Associated with Progression-Free Survival of Peripheral Neuroblastic Tumor Patients
Source: Cancers (Basel). 2025 Apr 12;17(8):1303. doi: 10.3390/cancers17081303 (PMC12025499; doi:10.3390/cancers17081303)
Supplement: Supplementary file 1 [file cancers-17-01303-s001.zip › cancers-3528567-supplementary.pdf]

Supplementary Material

Tertiary lymphoid structures are associated with progression-free survival of peripheral neuroblastic tumor patients

Rebecca Rothe [1,2,3,†], Therés Golle [1,†], Basma Hachkar [1], Tina Hörz [4], Jessica Pablik [5], Luise Rupp [1], Ina Dietsche [1], Christian Kruppa [4], Guido Fitze [4], Marc Schmitz [1,2,3], Michael Haase [4,‡] and Rebekka Wehner [1,2,3,*,‡]

1 Institute of Immunology, Faculty of Medicine Carl Gustav Carus, TUD Dresden University of Technology, 01307 Dresden, Germany; rebecca.rothe@nct-dresden.de (R.R.); theres.golle@tu-dresden.de (T.G.); basma.hachkar@uk-koeln.de (B.H.); luise.rupp@tu-dresden.de (L.R.); ina.dietsche@mailbox.tu-dresden.de (I.D.); marc.schmitz@tu-dresden.de (M.S.); rebekka.wehner@tu-dresden.de (R.W.)

2 National Center for Tumor Diseases (NCT), NCT/UCC Dresden, a partnership between DKFZ, Faculty of Medicine and University Hospital Carl Gustav Carus, TUD Dresden University of Technology, and Helmholtz-Zentrum Dresden-Rossendorf (HZDR), 01307 Dresden, Germany.

3 German Cancer Consortium (DKTK), 01307 Dresden, Germany.

4 Department of Pediatric Surgery, University Hospital Carl Gustav Carus, 01307 Dresden, Germany; tina.hoerz@ukdd.de (T.H.); christian.kruppa@ukdd.de (C.K.); guido.fitze@ukdd.de (G.F.); michael.haase@ukdd.de (M.H.)

5 Department of Pathology, University Hospital Carl Gustav Carus, 01307 Dresden, Germany; jessica.pablik@ukdd.de

* Correspondence: rebekka.wehner@tu-dresden.de

† These authors contributed equally to this work.

‡ These authors also contributed equally to this work.

1 Supplementary Figures

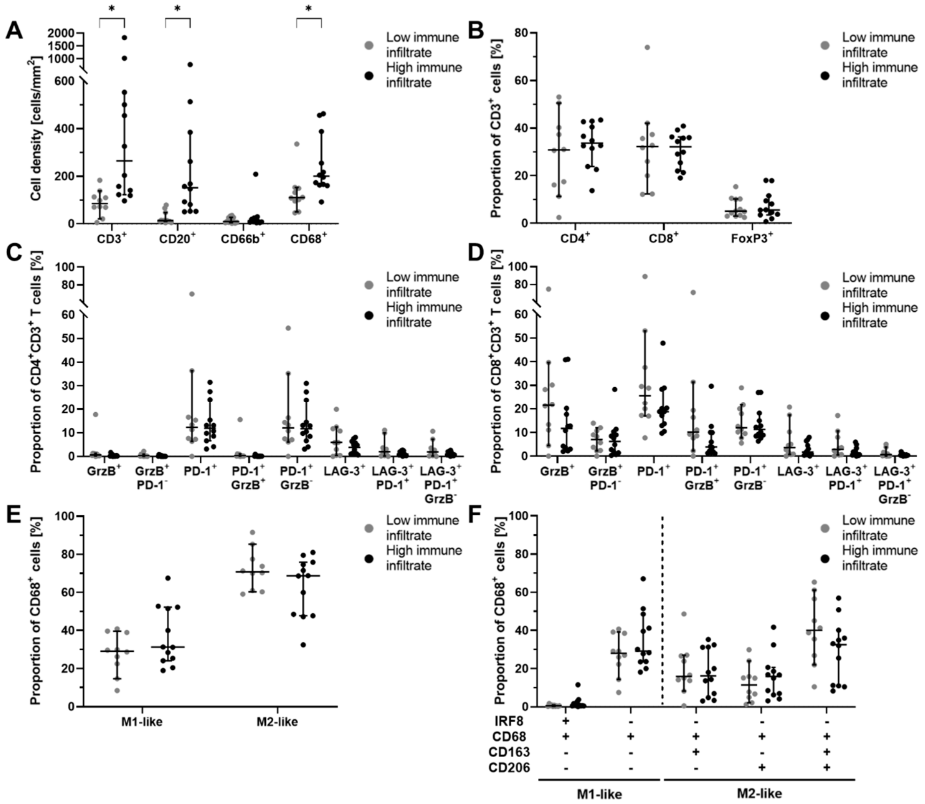

Figure S1. Immune cell frequencies in pNT patients clustered according to low and high immune cell infiltration. Cell densities of (**A**) CD3+, CD20+, CD66b+, and CD68+, (**B**) proportions of CD4+, CD8+ as well as FoxP3+ CD3+ T cells, (**C-D**) CD4+ CD3+ and CD8+ CD3+ T cells (co-)expressing GrzB, PD-1, and LAG-3, (**E**) M1- and M2-like macrophage proportions, and (**F**) divided M1- and M2-like macrophage populations are displayed as dot plots for low and high immune infiltrate groups. Positive marker expression (+) or absent marker expression (-) were defined for each investigated marker as displayed below the axes. Median with 95% confidence interval (CI); Mann-Whitney test; *$p \leq 0.05$.

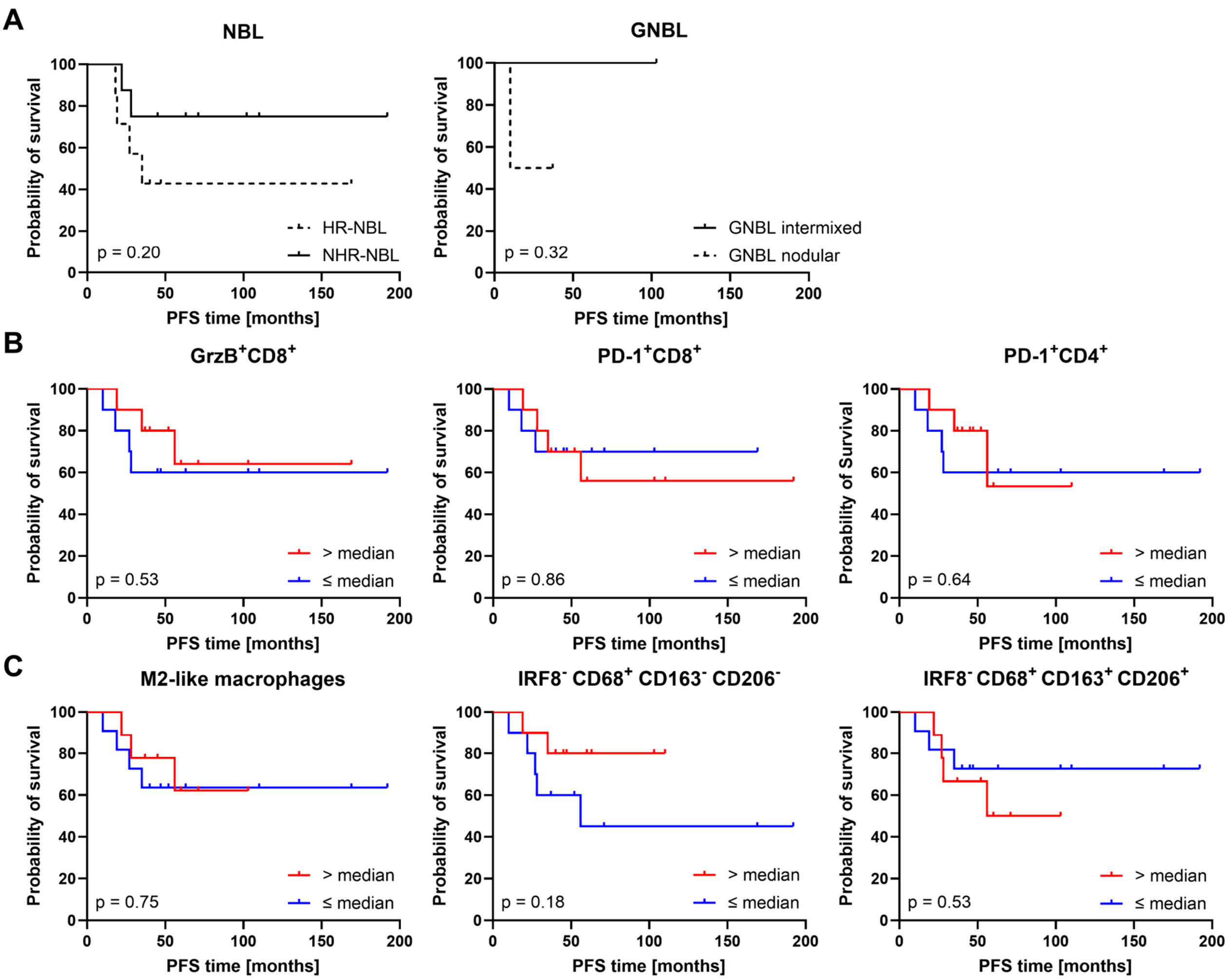

Figure S2: Survival analysis of individual T cell phenotypes and macrophage subpopulations. Kaplan-Meier plots depict progression-free survival (PFS) of (**A**) HR-NBL and NHR-NBL patients (left) as well as intermixed GNBL and nodular GN patients (right). PFS of all pNT patients is shown in Kaplan-Meier plots separating pNT patients according to the median frequencies of (**B**) $GrzB^+CD8^+$ (left), $PD\text{-}1^+CD8^+$ (center), and $PD\text{-}1^+CD4^+$ T cells (right) as well as (**C**) M2-like macrophages (left; summarizing $CD68^+CD163^+$, $CD68^+CD206^+$, and $CD68^+CD163^+CD206^+$ subpopulations), single $CD68^+$ (center), and triple $CD68^+CD163^+CD206^+$ macrophage subpopulations (right). *p*-values calculated by Log-rank test.

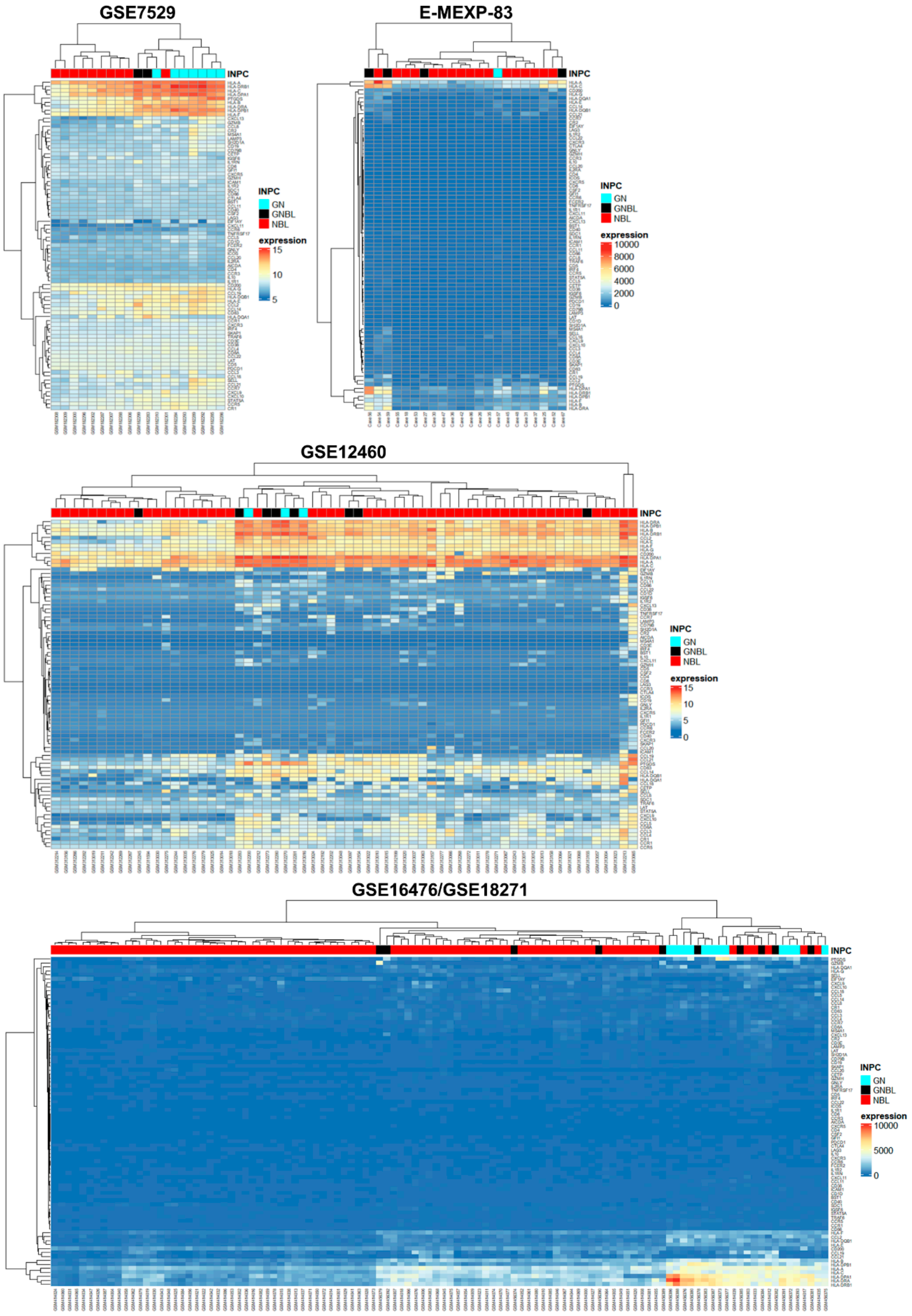

Figure S3: Heatmaps depict gene expression data of 83 TLS-related genes for four publically available microarray datasets using Euclidean distance for clustering columns and rows.

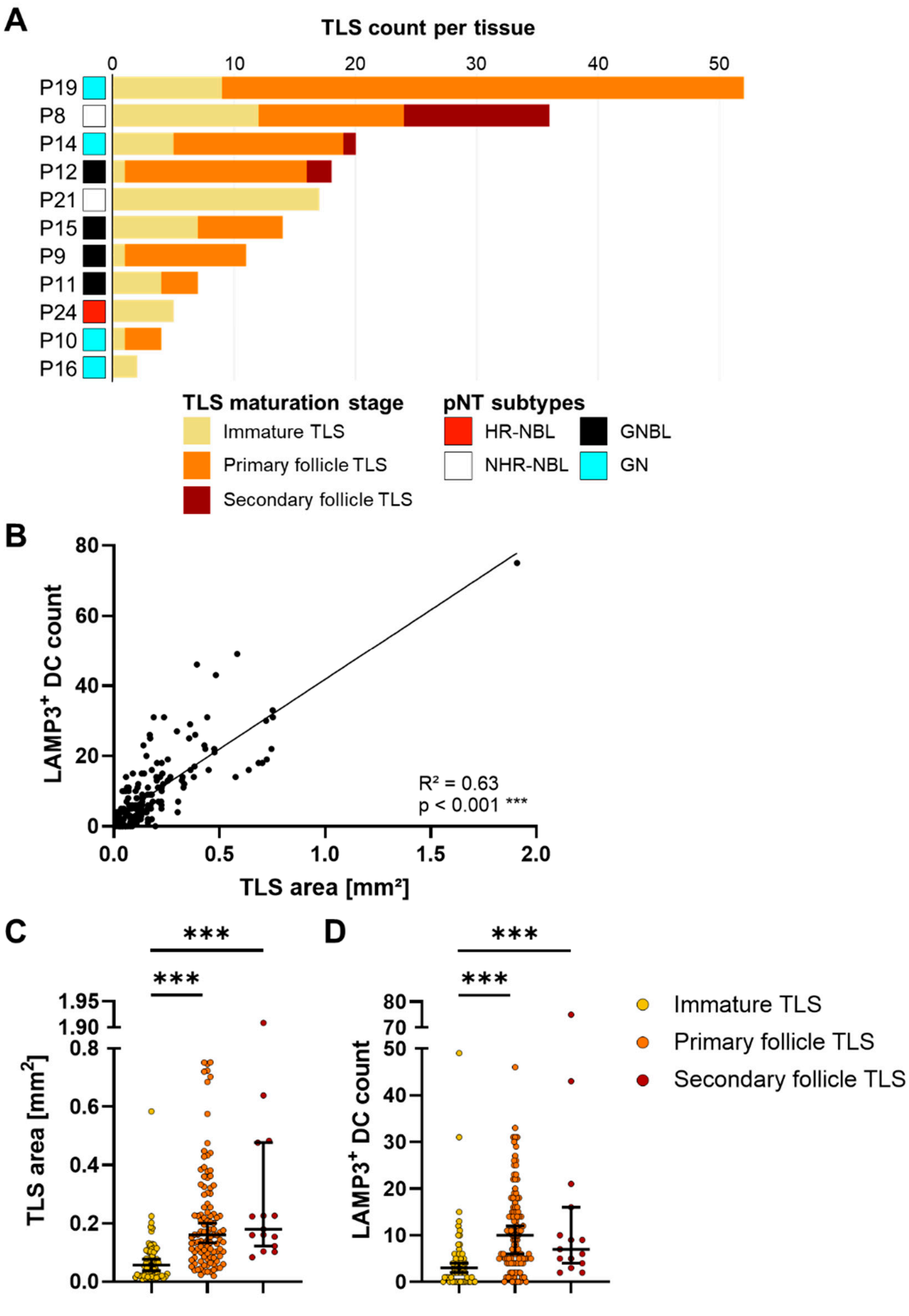

Figure S4: Analysis of TLS count per tissue and LAMP3+ DC in single TLS. (**A**) TLS count per tissue for all 11 TLS+ pNT patients with respect to TLS maturation stages (immature TLS, primary follicle TLS, and secondary follicle TLS) and pNT subtypes. (**B**) Linear regression of TLS area [mm^2] to LAMP3+ DC count within individual TLS. (**C**) Area of immature, primary follicle, and secondary follicle TLS. (**D**) Count of LAMP3+ DC in single immature, primary follicle, and secondary follicle TLS. Median with 95% confidence interval (CI); Mann-Whitney test; ***$p < 0.001$.

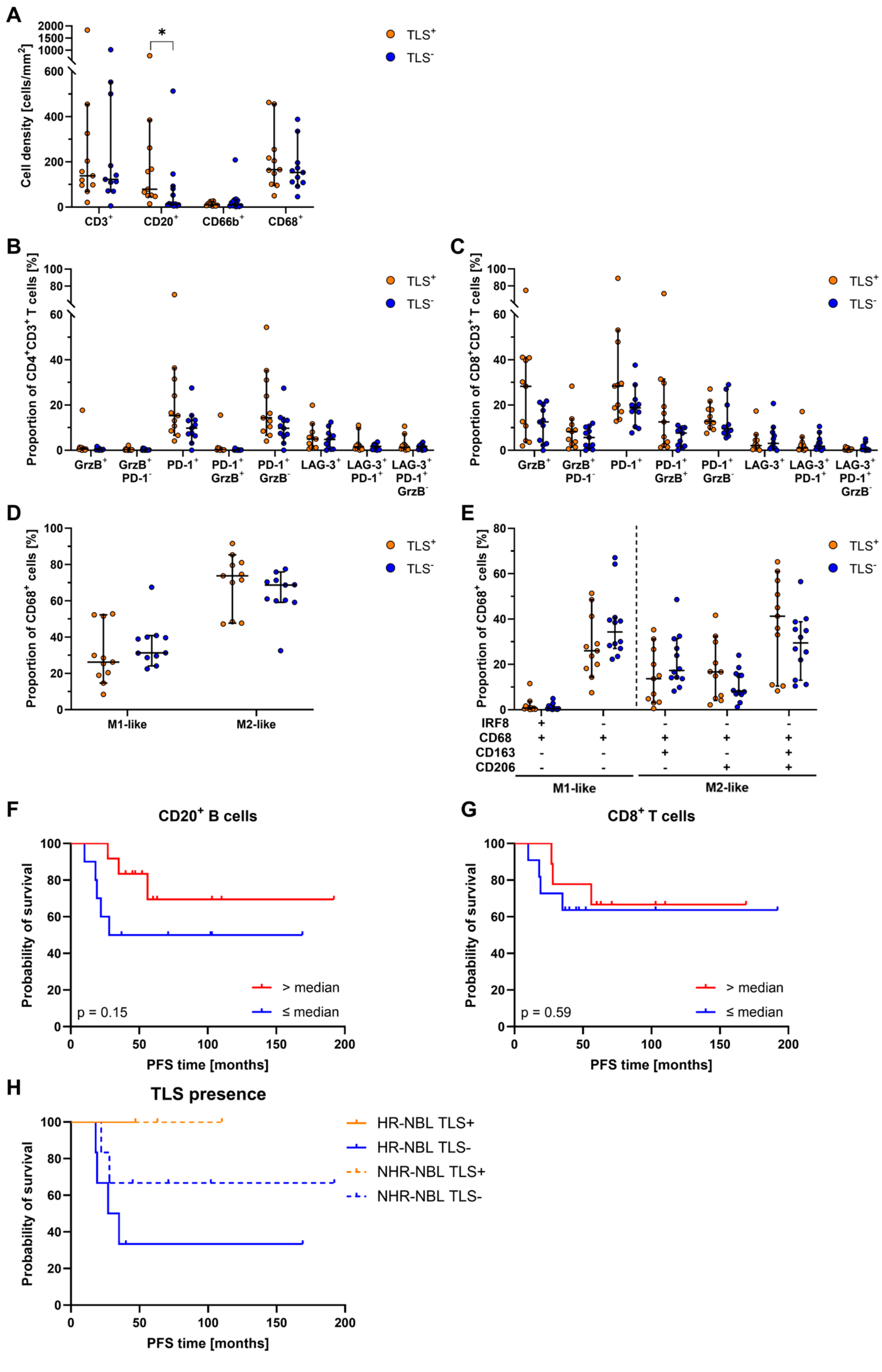

Figure S5: Immune cell frequencies based on TLS presence/absence in pNT tissue samples. TLS+ and TLS- tissues were compared in terms of (**A**) CD3+, CD20+, CD66b+, and CD68+ cell densities as well as (**B**-**C**) proportions of CD4+ CD3+ (left) and CD8+ CD3+ T cells (right) (co-)expressing GrzB, PD-1, and LAG-3, (**D**) M1- and M2-like macrophage proportions, and (**E**) divided M1- and M2-like macrophage populations. Positive marker expression (+) or absent marker expression (-) were defined for each investigated marker as displayed below the axes. Median with 95% confidence interval (CI). Kaplan-Meier curves show PFS of pNT patients separated according to the median frequencies of (**F**) CD20+ B cells and (**G**) CD8+ T cells. (**H**) Kaplan-Meier curves present PFS of TLS+ and TLS- HR-NBL as well as NHR-NLB patients. *p*-values calculated by Log-rank test.

2 Supplementary Tables

Table S1. List of 19 antibodies and reagents used for multiplex IHC.

Staining protocol	Target (human)	Host species	Dilution	Manufacturer	Order number	Clone	Incubation conditions	Opal dye	Dilution	Incubation conditions
**Overall immune cell infiltrate**	CD3	rabbit	1:300	Agilent Dako	A0452	polyclonal	32 min, 36°C	620	1:300	8 min, 36°C
	CD20	mouse	1:200	Invitrogen	14-0202-82	L26	32 min, 36°C	650	1:150	8 min, 36°C
	CD66b	mouse	1:50	BD Biosciences	555723	G10FS	32 min, 36°C	690	1:50	8 min, 36°C
	CD68	rabbit	1:250	Cell Signalling Technology	76437	D4B9C	32 min, 36°C	520	1:150	8 min, 36°C
	FoxP3	mouse	1:50	Abcam	ab20034	236A/E7	32 min, 36°C	540	1:100	8 min, 36°C
	CD56 (TM)	rabbit	1:100	Cell Signalling Technology	99746T	E7X9M	32 min, 36°C	570	1:1000	8 min, 36°C
	synaptophysin (TM)	rabbit	1:1000	Cell Marque	336R	MRQ-40	32 min, 36°C	570	1:1000	8 min, 36°C
**Macro-phages**	CD68	rabbit	1:100	Cell Signalling Technology	76437	D4B9C	32 min, RT	520	1:100	8 min, 36°C
	CD163	mouse	pre-diluted	Roche	5973929001	MRQ-26	32 min, 36°C	540	1:250	8 min, 36°C
	CD206	rabbit	1:75	Cell Signalling Technology	91992	E2L9N	32 min, 36°C	690	1:50	8 min, 36°C
	IRF8	mouse	1:400	Santa Cruz Biotechnology	sc-365042	E-9	32 min, 36°C	570	1:800	8 min, 36°C
**T cells**	CD3	rabbit	pre-diluted	Roche	5278422001	2GV6	16 min, RT	690	1:100	8 min, 36°C
	CD4	rabbit	1:50	Abcam	ab81289	EPR6855	32 min, 36°C	520	1:75	8 min, 36°C
	CD8	mouse	1:100	Agilent Dako	M7103	C8/144B	32 min, 36°C	540	1:100	8 min, 36°C
	Granzyme B	mouse	1:50	Agilent Dako	M7235	Grb-7	32 min, 36°C	570	1:50	8 min, 36°C
	LAG-3	rabbit	1:50	Cell Signalling Technology	15372	D2G40	32 min, 36°C	650	1:50	8 min, 36°C
	PD-1	mouse	pre-diluted	Roche	7099029001	NAT105	60 min, 36°C	620	1:50	8 min, 36°C
**TLS**	CD3	rabbit	pre-diluted	Roche	5278422001	2GV6	32 min, 36°C	520	1:75	8 min, 36°C
	CD20	mouse	1:400	Invitrogen	14-0202-82	L26	32 min, 36°C	650	1:700	8 min, 36°C
	Ki67	mouse	1:75	Agilent Dako	M7240	MIB-1	32 min, 36°C	690	1:75	8 min, 36°C
	LAMP3	rabbit	1:500	Cell Signalling Technology	47778	E6E5U	32 min, 36°C	620	1:750	8 min, 36°C
	PNAd*	rat*	1:400	BioLegend	120801	MECA-79	32 min, 36°C	570	1:500	8 min, 36°C
	CD56 (TM)	rabbit	1:50	Cell Signalling Technology	99746T	E7X9M	32 min, 36°C	540	1:400	8 min, 36°C
	chromogranin A (TM)	mouse	1:250	Sigma-Aldrich	238M	LK2H10	32 min, 36°C	540	1:400	8 min, 36°C
	synaptophysin (TM)	rabbit	1:500	Cell Marque	336R	MRQ-40	32 min, 36°C	540	1:400	8 min, 36°C

*used with a bridging Ab (rabbit anti-rat IgG, polyclonal, Vector laboratories, order number AI-40017). TM – tumor marker

Table S2. List of TLS-related genes.

AICDA	CD19	CXCL11	HLA-DRB1	SDC1
BST1	CD1D	CXCL13	HLA-E	SELL
CCL11	CD200	CXCL9	HLA-F	SH2D1A
CCL14	CD38	CXCR3	HLA-G	SKAP1
CCL18	CD3E	CXCR5	ICAM1	STAT5A
CCL19	CD4	EIF1AY	ICOS	TNFRSF17
CCL2	CD40	FCER2	IGSF6	TRAF6
CCL20	CD5	GFI1	IL10	
CCL21	CD6	GNLY	IL1R1	
CCL22	CD79B	GZMB	IL1R2	
CCL3	CD83	GZMH	IL1RN	
CCL4	CD86	HLA-A	IL2RA	
CCL5	CD8A	HLA-B	IRF4	
CCL8	CETP	HLA-C	LAG3	
CCR1	CR1	HLA-DPA1	LAMP3	
CCR3	CR2	HLA-DPB1	LAT	
CCR5	CSF2	HLA-DQA1	MS4A1	
CCR6	CTLA4	HLA-DQB1	PDCD1	
CCR7	CXCL10	HLA-DRA	PTGDS	
